# Prognostic and immune infiltration signatures of proteasome 26S subunit, non-ATPase (PSMD) family genes in breast cancer patients

**DOI:** 10.18632/aging.203722

**Published:** 2021-11-28

**Authors:** Do Thi Minh Xuan, Chung-Che Wu, Tzu-Jen Kao, Hoang Dang Khoa Ta, Gangga Anuraga, Vivin Andriani, Muhammad Athoillah, Chung-Chieh Chiao, Yung-Fu Wu, Kuen-Haur Lee, Chih-Yang Wang, Jian-Ying Chuang

**Affiliations:** 1 Graduate Institute of Cancer Biology and Drug Discovery, College of Medical Science and Technology, Taipei Medical University, Taipei 11031, Taiwan; 2 Division of Neurosurgery, Department of Surgery, School of Medicine, College of Medicine, Taipei Medical University, Taipei 11031, Taiwan; 3 Division of Neurosurgery, Department of Surgery, Taipei Medical University Hospital, Taipei 11031, Taiwan; 4 The Ph.D. Program for Neural Regenerative Medicine, Taipei Medical University, Taipei 11031, Taiwan; 5 Ph.D. Program for Cancer Molecular Biology and Drug Discovery, College of Medical Science and Technology, Taipei Medical University and Academia Sinica, Taipei 11031, Taiwan; 6 Department of Statistics, Faculty of Science and Technology, PGRI Adi Buana University, Surabaya 60234, East Java, Indonesia; 7 Department of Biological Science, Faculty of Science and Technology, Universitas PGRI Adi Buana, Surabaya 60234, East Java, Indonesia; 8 Department of Medical Research, Tri-Service General Hospital, School of Medicine, National Defense Medical Center, Taipei 11490, Taiwan; 9 Cancer Center, Wan Fang Hospital, Taipei Medical University, Taipei 11031, Taiwan; 10 TMU Research Center of Cancer Translational Medicine, Taipei Medical University, Taipei 11031, Taiwan; 11 Department of Biomedical Science and Environmental Biology, Kaohsiung Medical University, Kaohsiung 80708, Taiwan; 12 Cell Physiology and Molecular Image Research Center, Wan Fang Hospital, Taipei Medical University, Taipei 11031, Taiwan

**Keywords:** PSMD family genes, bioinformatics, breast cancer

## Abstract

The complexity of breast cancer includes many interacting biological processes that make it difficult to find appropriate therapeutic treatments. Therefore, identifying potential diagnostic and prognostic biomarkers is urgently needed. Previous studies demonstrated that 26S proteasome delta subunit, non-ATPase (PSMD) family members significantly contribute to the degradation of damaged, misfolded, abnormal, and foreign proteins. However, transcriptional expressions of PSMD family genes in breast cancer still remain largely unexplored. Consequently, we used a holistic bioinformatics approach to explore PSMD genes involved in breast cancer patients by integrating several high-throughput databases, including The Cancer Genome Atlas (TCGA), cBioPortal, Oncomine, and Kaplan-Meier plotter. These data demonstrated that PSMD1, PSMD2, PSMD3, PSMD7, PSMD10, PSMD12, and PSMD14 were expressed at significantly higher levels in breast cancer tissue compared to normal tissues. Notably, the increased expressions of PSMD family genes were correlated with poor prognoses of breast cancer patients, which suggests their roles in tumorigenesis. Meanwhile, network and pathway analyses also indicated that PSMD family genes were positively correlated with ubiquinone metabolism, immune system, and cell-cycle regulatory pathways. Collectively, this study revealed that PSMD family members are potential prognostic biomarkers for breast cancer progression and possible promising clinical therapeutic targets.

## INTRODUCTION

According to statistical data of cancer incidence and mortality, breast cancer (BRCA) accounts for 30% of newly diagnosed cases of cancer among American women [1, 2]. The currently used stratification system is still undergoing changes due to the heterogeneity of this disease, which can be observed at both the molecular and histological levels. Based on the presence or absence of prevalent listed biomarkers, including: the estrogen receptor (ER), progesterone receptor (PR), human epidermal growth factor receptor (HER)-2, and some other markers. Stratifying BRCA not only helps in selecting treatment options but also assists in approximating treatment responses and predicting prognostic statuses.

Many different treatment strategies besides surgery are available for patients with BRCA. Treatment options are personalized and often based on a multi-modality approach, depending on several factors, including the stage and biology of the tumor (hormone receptor and nodal status); genomic markers (Oncotype DX™ or MammaPrint™) [3, 4]; patient age, physical condition, menopausal status, and the presence of inherited genetic mutations (such as BRCA1 or BRCA2); and a patient’s acceptance and tolerance of treatment regimens. Some treatments are standard, such as surgical therapy, radiotherapy, systemic therapy (endocrine therapy, chemotherapy, and targeted therapy), and immuno-therapy, while others are undergoing clinical trials. As one of the potential approaches, targeted therapies are selective inhibitors which only affect altered cancer cells [5, 6]. They precisely identify and attack specific molecules to block cancer growth, progression, and metastasis. Most targeted therapies are either monoclonal antibodies (mAbs) or small-molecule drugs (tyrosine kinase inhibitors, cyclin-dependent kinase inhibitors, poly (ADP-ribose) polymerase (PARP) inhibitors) and mammalian target of rapamycin (mTOR) inhibitors [7–9]. Nevertheless, drugs resistance which may develop soon after onset of this therapy is the main challenge to current research. Meanwhile, immunotherapeutic strategies, which are drugs designed to strengthen the body's natural defenses to fight cancer, have appreciably raised our expectations of successfully treating various cancer types [10–15]. In general, immunotherapies are further categorized into various subtypes, such as mAbs, immune checkpoint blockade (anti-cytotoxic T-lymphocyte-associated (CTLA)-4, anti-programmed death (PD)-1, anti-PD-ligand 1 (L1)), cytokine therapy, T-cell transfer therapy (including tumor-infiltrating lymphocytes (or TIL) therapy and chimeric antigen receptor (CAR) T Cell Therapy), and therapeutic vaccines. For instance, the immune checkpoint inhibitors that target the PD-1 pathway (pembrolizumab, atezolizumab, dostarlimab) are approved by the US Food and Drug Administration (FDA) for patients with metastatic TNBC [16–21]. According to recent literature, the abovementioned treatments for early BRCA determined by sub-classification have significantly improved the prognosis of BRCA patients with a 5-year survival rate of more than 85%. Therefore, it is crucial for us to understand the occurrence and development of breast cancer and to find biomarkers that indicates the sensitivity of current therapies and long-term outcomes in the early stage of the disease [22–28].

The ubiquitin-proteasome system is an indispensable mechanism of highly regulated intracellular protein degradation and turn over, thus dominates human antigen processing, signal transduction and cell-cycle regulation. The 26S proteasome is composed of one proteolytically active cylinder-shaped particle (the 20S proteasome), and one or two ATPase-containing complexes (known as the 19S cap complexes). The 20S core is constructed from inner α-rings and outer β-rings, which are both divided into 7 structurally similar subunits: proteasome 20S subunit α (PSMA1~7) and β (PSMB1~7), respectively. The 19S cap complexes is composed of a base and a lid subcomplex, further categorized into ATPase subunits (PSMC1~6) and non-ATPase subunits (PSMD1~14) [29–33]. In recent studies, dysfunction of the ubiquitin-proteasome system, which manifests as up- and/or downregulation of the aforementioned genes, has been described in various oncogenic situations. Hence, extensive research need to be conducted to fully assess the oncogenic potential of this family genes.

The PSMD family, which is comprised of 14 members in total, was proven to be partially involved in the formation of the regulatory complex. Both components occupy an important place in modulating the proteasome that performs several essential functions, such as catalyzing the unfolding and translocation of substrates into the 20S proteasome. Recent studies showed that *PSMD1* and *PSMD3* act as oncogenes in chronic myeloid leukemia by stabilizing nuclear factor (NF)-κB [34]. In gastric cancer, interactions between PSMD2 and asporin induced cell proliferation [35]. PSMD4 influenced cell malignancy of esophageal cancer via suppressing endoplasmic reticular (ER) stress [36]. PSMD5 inactivation promoted 26S proteasome assembly during colorectal tumor progression [37]. PSMD6, PSMD9, PSMD11, and PSMD14 expressions were significantly related to decreased survival chances in pancreatic ductal adenocarcinoma [38]. High-throughput technologies are widely used as systematic approaches to explore differences in expressions of thousands of genes in both biological and genomics systems [39–41]. Abnormal gene expressions are generally related to oncogenes and tumor-suppressor genes which regulate tumor maturation [42–47].

However, no studies have yet been conducted to develop data of how messenger (m)RNA levels of each *PSMD* family gene change in BRCA development. Therefore, this study aimed to make relevant comparisons of gene expressions in BRCA and normal tissues, by extracting information from public datasets, including numerous RNA-sequencing (RNA-Seq) and microarrays data of BRCA patients.

Moreover, we also explored the interactive cooperation or gene regulatory networks in which the targeted family genes were involved to identify completely novel biomarkers [48–53]. By adopting a meta-analytical approach, downstream molecules associated with *PSMD* genes were effectively screened. The study findings revealed that these PSMD family members and their regulated gene counterparts are worth considering as novel therapeutic targets for BRCA patients.

## RESULTS

### PSMD family members are involved in important processes in the developmental stages of BRCA

**Figure 1:**
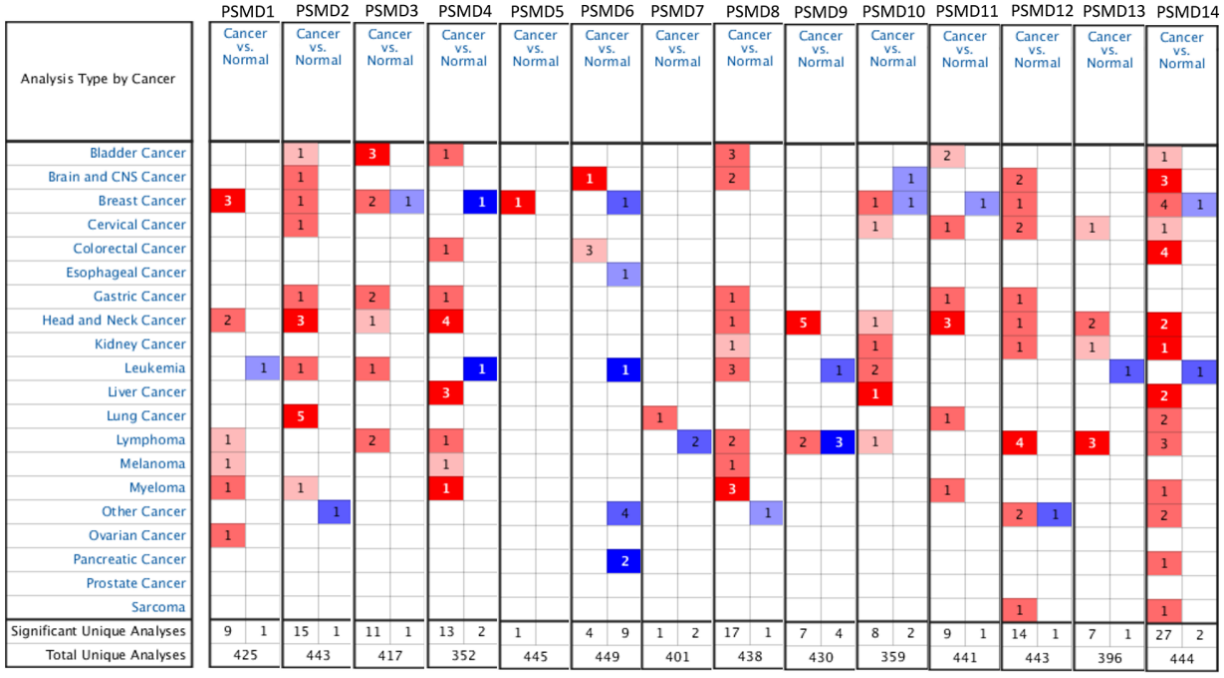
**Systemic analysis of 26S proteasome delta subunit, non-ATPase (*PSMD*) family genes in 20 common types of cancer (Oncomine platform).** Dysregulation of each *PSMD* individual gene in targeted cancer tissues as measured by the mRNA expression level was compared to their normal counterparts using Students’ *t*-test. The cutoff parameters were set as follows: *p*<0.05; multiple of change >2; and gene rank in the top 10%. The quantity of datasets which met those thresholds was represented as a number inside the table cells, while colors (red or blue) indicate the trend of gene expressions (up- or downregulation, respectively) and the intensity of colors indicates the degree of abnormal expression.

Prior studies discovered PSMD family members in human and significant roles in cancer progression of some of them. To provide further identification of *PSMD* family gene signatures related to breast neoplasms, a meta-analysis was carried out. As reported by an Oncomine analysis of mRNA expressions among PSMD family members, including PSMD1, PSMD2, PSMD3, PSMD5, PSMD10, PSMD12, and PSMD14 are highly upregulated in BRCA tissues. It was suggested that their overexpression promotes tumor growth. Therefore, we decided to perform further bioinformatics analyses on BRCA (Figure 1). Since the Kaplan-Meier curves are univariate analysis, the univariate and multivariate Cox proportional hazards regression analysis, which works for both quantitative predictor variables and for categorical variables, was subsequently verified by TCGA-based breast cancer samples. Results was presented in Supplementary Table 1.

### Associations of *PSMD* family gene interpretations in neoplastic cell lines with clinicopathological parameters of BRCA patients

**Figure 2:**
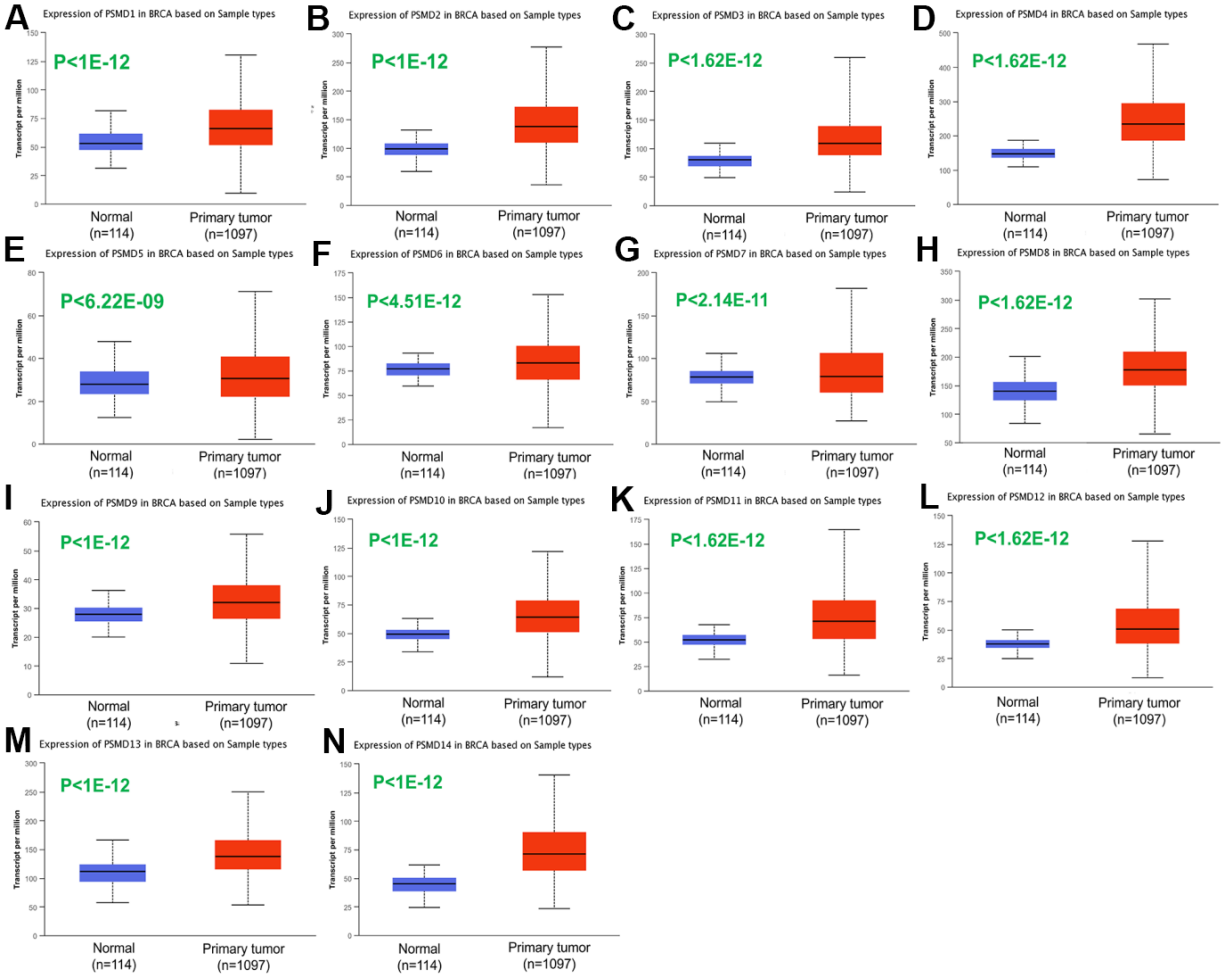
**Transcriptional expression of 26S proteasome delta subunit, non-ATPase (PSMD) family members in breast cancer (BRCA) patients.** (**A**–**N**) Transcriptome alterations observed in PSMD1~14. Boxplot of PSMD mRNA expression levels measured in BRCA specimens (red) compared to their normal counterparts (blue) obtained from the UALCAN database. Statistical analysis was performed using Student’s *t*-test, and p<0.05 was considered statistically significant.

**Figure 3:**
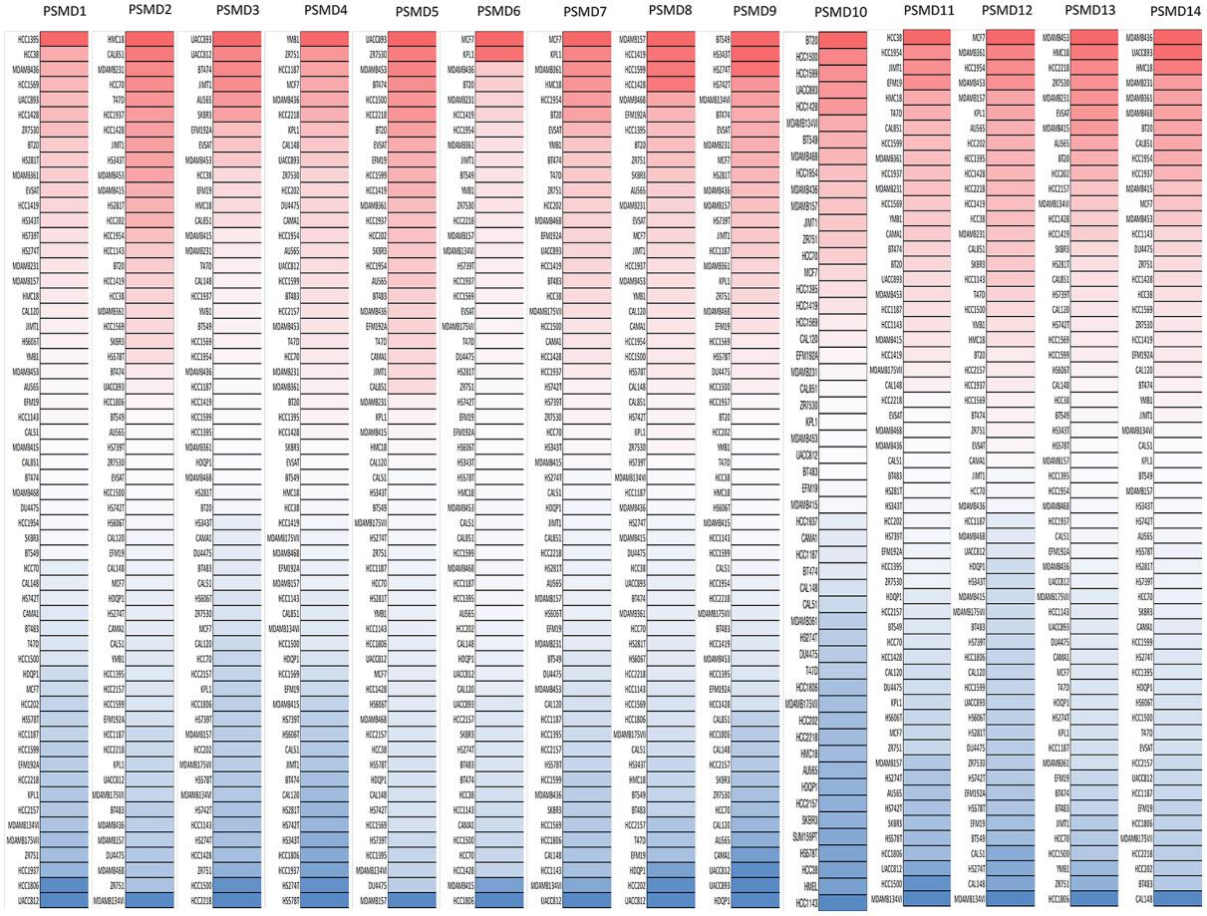
**Expressions of 26S proteasome delta subunit, non-ATPase (*PSMD*) genes measured in common types of breast cancer (BRCA) cell lines.** A CCLE database-built heatmap plot presents patterns of changes in expression levels of *PSMD* family genes among different BRCA cell lines. Shades of colors vary from red (overexpressed sample) to white (no change in gene expressions) and blue (under-expressed sample). The darker the colors are, the higher the gene expressions that were recorded.

After properly examining differences in *PSMD* family gene expressions between neoplastic and normal tissues using GEPIA2 datasets, we found that all mRNA levels of the former were upregulated compared to the latter, with the *q*-value cutoff set to <<0.001 (Figure 2). In addition, analysis performed on a Cancer Cell Line Encyclopedia (CCLE) dataset (https://www.broadinstitute.org/ccle) also indicated that PSMD mRNA levels were overexpressed in BRCA tissues (Figure 3).

### Analysis of genes related to BRCA co-expressed with *PSMD* family genes

**Figure 4:**
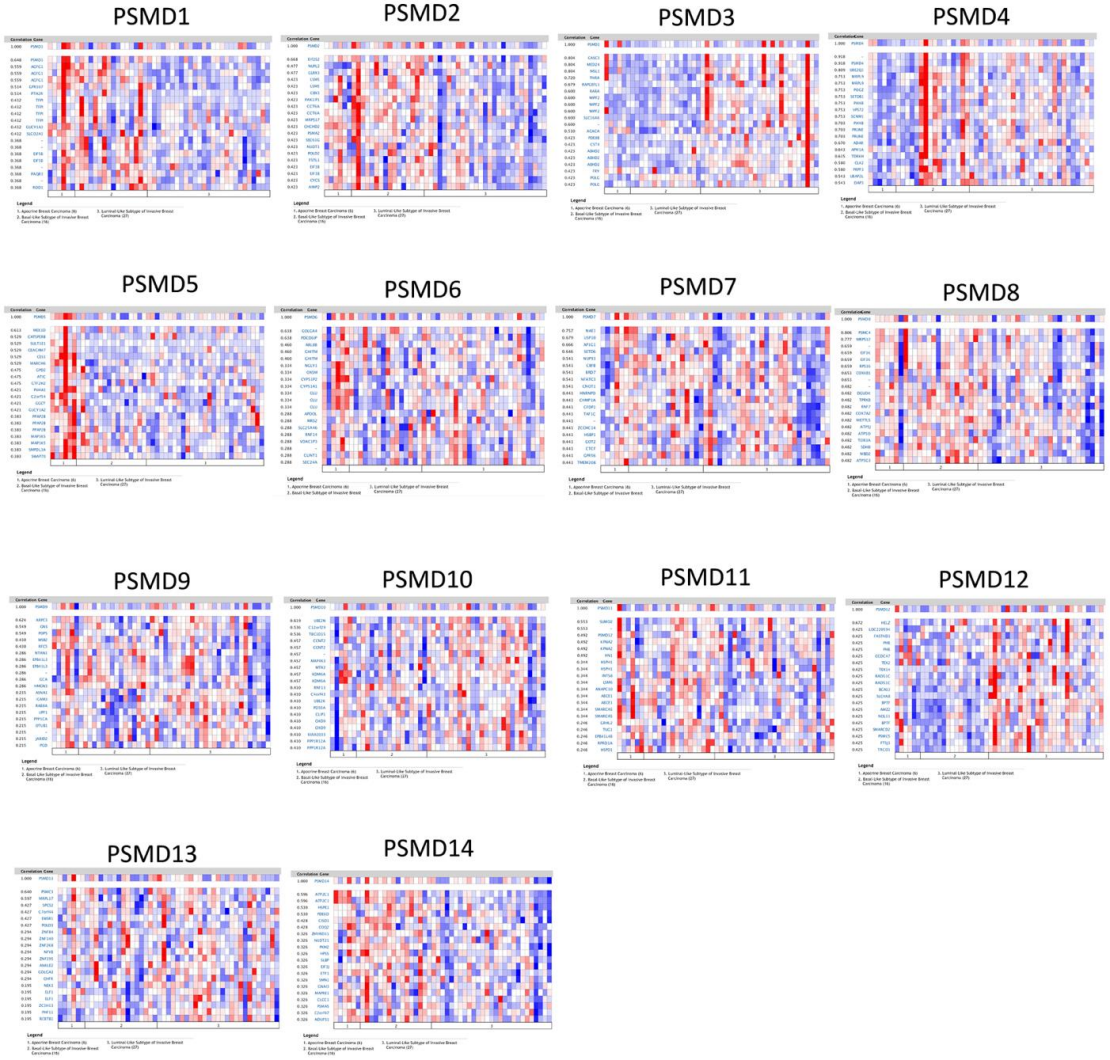
**Heatmap co-expression profiles of 26S proteasome delta subunit, non -ATPase (PSMD) family members in breast cancer (BRCA).** Genes co-expressed with each of the PSMD family members in term of BRCA patients are presented in a heatmap format (data extracted from the Oncomine database).

By leveraging the Oncomine online platform to perform a thorough analysis of the co-expression network of *PSMD1*, we found that *PSMD1* was positively correlated with *AGFG1*, *GPR107*, *PTH2R*, *TFPI*, *GUCY1A3*, *SLCO2Al*, *EIF5B*, *PAQR3*, and *ROD1*. As for genes which are supposedly co-expressed with *PSMD2*, we concluded that its expression was positively correlated with *EIF2S2*, *NUPL2*, *GLRX3*, *LSM5*, *CBX3*, *PAKIIP1*, *CCT6A*, *MRPS17*, *CHCHD2*, *PSMA2*, *SEC61G*, *NUDT1*, *POLD2*, *FSTL1*, *EIF3B*, *CYCS*, and *AIMP2*. As for genes co-expressed with *PSMD3*, there were positive correlations with *CASC3*, *MED24, MSL1, THRA, RAPGEFL1, RARA, WIPF2, SLC16A6, ACACA, PDESB, CST4, ABHD2, FRY*, and *POLG*. Similarly, genes co-expressed with *PSMD4* included *UBE2Q1, MRPL9, POGZ, SETDB1, P14KB, VPS72, SCNM1, P14KB, PRUNE, ADAR, APH1A, TDRKH, CLK2, PRPF3, UBAPZL*, and *DAP3*. Moreover, positive correlations with *PSMD5* were determined for *MEX3D, CATSPERB, SULT1E1, CEACAM7, CES1, MARCH6, GPD2, ATIC, GTF2H2, P4HAL, C2ORF54, GGCT, GUCY1A2, PPAP2B, MAP3K5, SMPDL3A*, and *SWAP70*. Similar to previous cases, *PSMD6* was found to be positively correlated with *GOLGA4, PDCD6IP, ARL8B, GHITM, NGLY1, OXSM, CYP51P2, CYP51A1, CLU, APOOL, MRS2, SLC25A46, RNF14, VDACIP3, CLINT1*, and *SEC24A*. We found that genes co-expressed with *PSMD7* included *NAE1, USP10, AP1G1, SETD6, NUP93, CBFB, BRD7, NFATC3, CNOTI, HNRNPD, CHMP1A, CFDP1, TAFIC, ZCCHC14, HSBP1, GOT2, CTCF, GPR56*, and *TMEM208*. Genes co-expressed with *PSMD8* included *PSMC4, MRPS12, EIF3K, EIF3K, RPS16, COX6B1, DGUOK, TPRKB, RNF7, COX7A2, METTL5, ATP5J, ATP50, TOR3A, SDHB, MBD2*, and *ATP5G3*. As for genes co-expressed with *PSMD9*, there were positive correlations with *ARPC3, GNS, POP5, WSB2, RFC5, NTAN1, EPB41L3, EPB41L3, GCA, HMGN3, ASNAI, ICAM3, RAB8A, UPF1, PPPICA, OTUBI, JARIDZ*, and *PGD*. Genes co-expressed with *PSMD10* included *UBEZN, C12orf29, TBC1D15, CCNT2, MAP4K3, MTX2, KDM6A, RNF13, C4orf43, UBE2K, PDS5A, CLIP1, CHD9, KIAA1033, PPPIR1ZA*, and *PPP1R12A*. Moreover, *PSMD11* was positively correlated with *SUMOZ, PSMD12, KPNA2, HN1, HSPH1, INTS8, LSM6, ANAPC10, ABCE1, ABCE1, SMARCA5, GRHL2, TUG1, EPB41L4B, RPRD1A*, and *HSPD1*. PSMD12 was found to be positively correlated with *HELZ, LOC220594, FASTKD3, PHB, CCDC47, TEX2, TEX14, RAD51C, BCAS3, SLC4A8, BPTF, AMZ2, NOL11, BPTF, SMARCD2, PSMC5, FTSJ3*, and *TACOI*. Genes co-expressed with *PSMD13* included *PSMC3, MRPL17, SPCS2, C7orf44, EWSR1, POLD3, ZNF84, ZNF140, ZNF268, NFYB, ZNF195, ANKLE2, GOLGA3, CHFR, NEK3, ELF1, ZC3H13, PHF11*, and *RCBTB1*. Finally, genes co-expressed with *PSMD14* were *ATP2C1, ATP2C1, HSPE1, PDE6D, CISD1, COQ2, ZMYND11, NUDT21, PKM2, HPS5, SLBP, EIF3J, ETF1, SMN1, GNAI3, MAPRE1, CLCC1, PSMA5, C2orf47*, and *NDUFS1* (Figure 4).

### Relationships between disease prognostication and *PSMD* gene expression levels measured in tumor specimens

**Figure 5:**
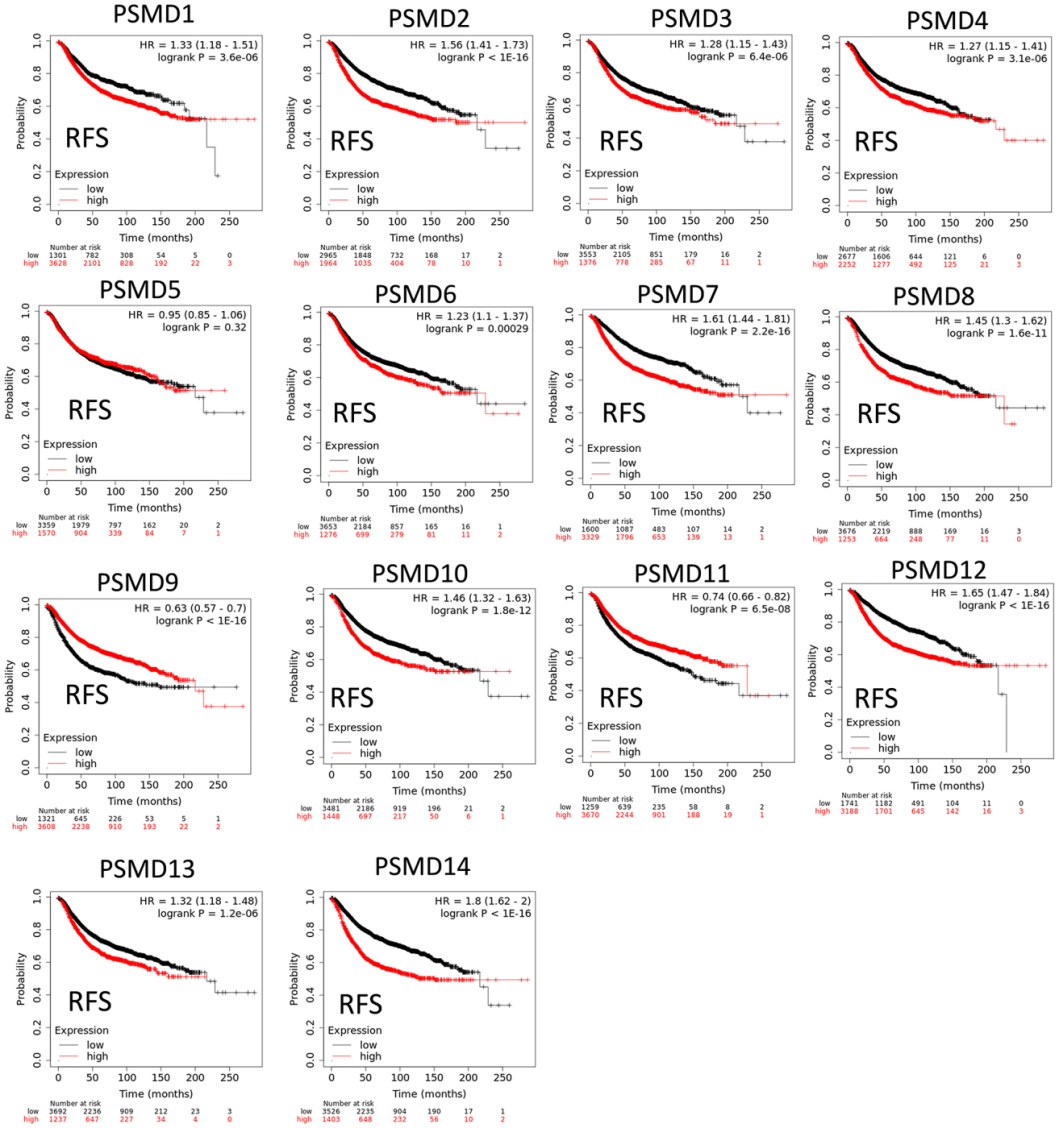
**Significant correlations between mRNA levels of 26S proteasome delta subunit, non-ATPase (PSMD) family members and recurrence-free survival curve (RFS) of patients diagnosed with breast cancer (BRCA).** The two survival curves respectively illustrate survival outcomes (including survival percentages and survival times) of BRCA patients with high (red) or low (black) expression levels of PSMD family members. Increased mRNA levels of target genes resulted in poor prognoses, while increasing levels of PSMD9 and PSMD11 were associated with favorable outcomes (*p*<0.05 was considered statistically significant).

**Figure 6:**
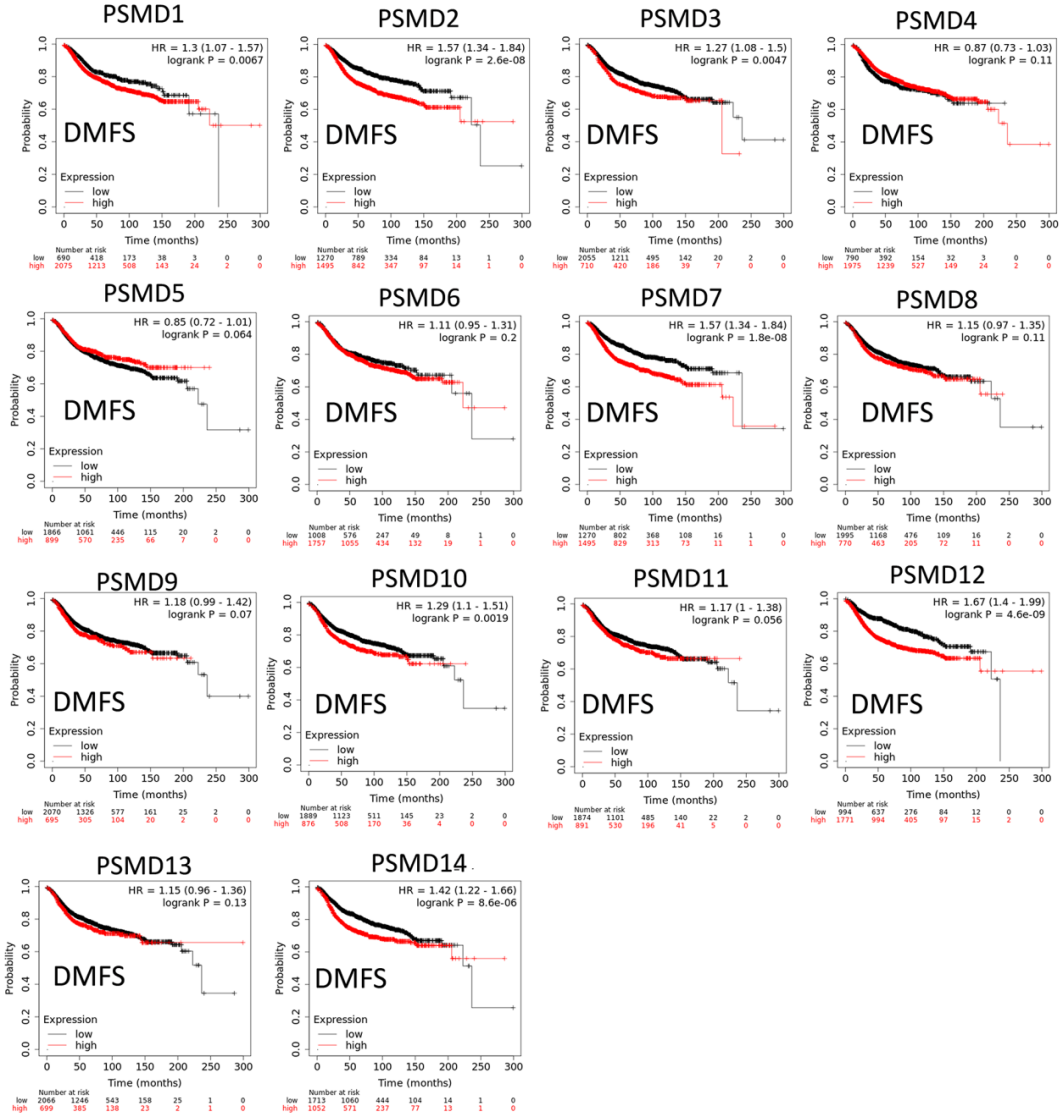
**Significant correlations between mRNA levels of 26S proteasome delta subunit, non-ATPase (PSMD) family members, and distant metastasis-free survival (DMFS) curve of patients diagnosed with breast cancer (BRCA).** The two survival curves respectively illustrate survival outcomes (including survival percentages and survival times) of BRCA patients with high (red) and low (black) expression levels of PSMD family members. Increased mRNA levels of target genes resulted in poor prognoses, except for PSMD4, PSMD5, PSMD6, PSMD8, PSMD9, PSMD11, and PSMD13 (*p*<0.05 was considered statistically significant).

**Figure 7:**
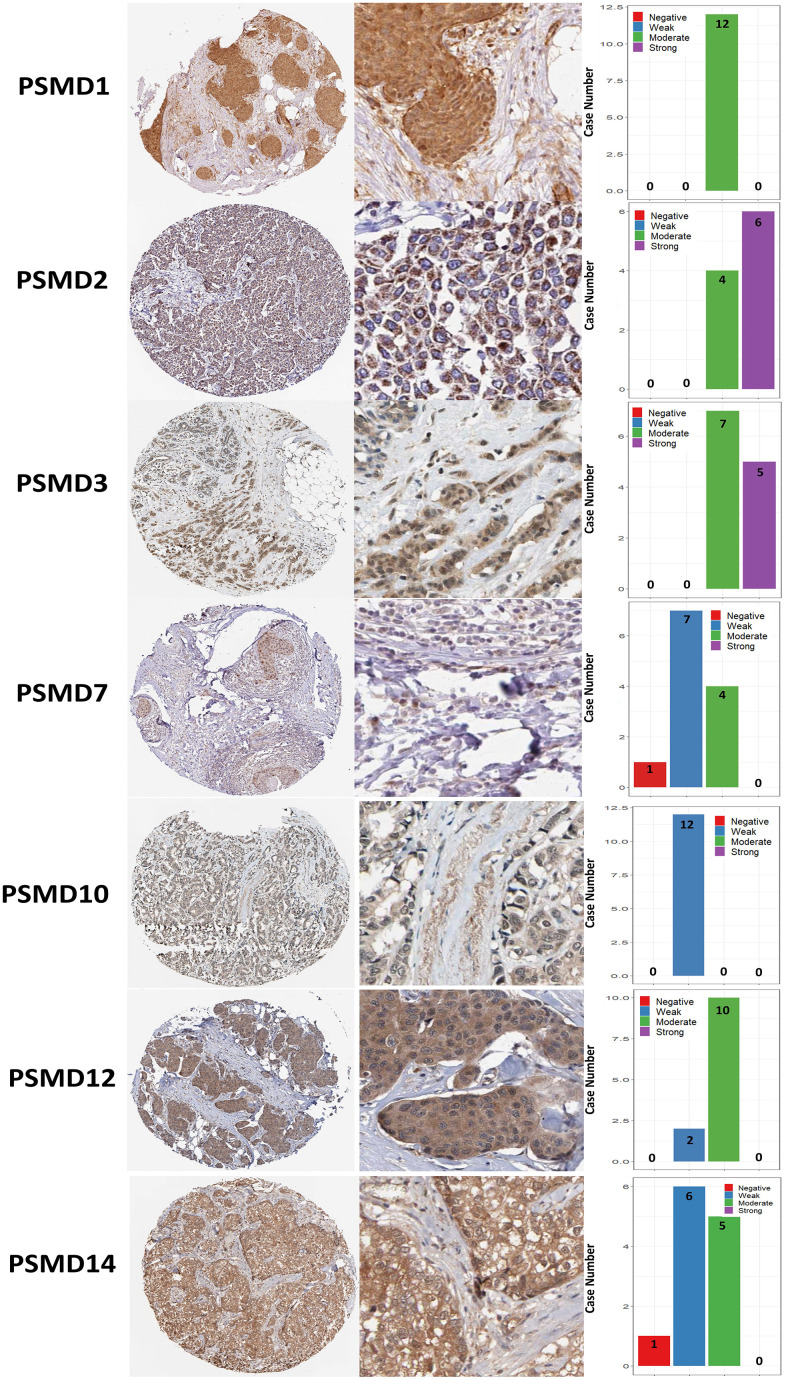
**Immunohistochemical staining of 26S proteasome delta subunit, non-ATPase (PSMD) family members in normal tissues and breast cancer (BRCA) tissues represented in IHC staining images and bar chart.** The images illustrate intensities of antibodies in both BRCA and adjacent normal tissues while the bar charts of IHC staining show intensities of PSMD family members in BRCA.

The Kaplan-Meier (KM) plotter database also indicated that most PSMD family members were associated with poor recurrence-free survival (RFS), except for PSMD9 and PSMD11. Higher expression levels of PSMD9 and PSMD11 were significantly associated with better survival rates of patients (Figure 5). We also validated these data from the NCBI GEO database (GSE21653) [54], and also obtained consistent data (Supplementary Figure 1). In addition, high expression levels of PSMD1, PSMD2, PSMD3, PSMD7, PSMD10, PSMD12, and PSMD14 were linked with poor distant metastasis-free survival (DMFS), whereas others were not (Figure 6). The RFS and DMFS data implied that these genes have oncogenic roles in BRCA progression. Therefore, we chose PSMD1, PSMD2, PSMD3, PSMD7, PSMD10, PSMD12, and PSMD14 as objectives for further bioinformatics analyses. Due to the fact that samples from BRCA patients displayed distinctly different expressions of *PSMD* family genes, we continued to explore how these target genes participate in particular metabolic pathways prior to investigating their clinical relevance. Therefore, the intensities of antibodies represented in clinical BRCA specimens were extracted from the Human Protein Atlas (HPA) for further analysis. Immunohistochemical (IHC) images revealed dense distributions of PSMD2 and PSMD4, while the other PSMDs, including PSMD1, PSMD2, PSMD3, PSMD7, PSMD12, and PSMD14, were moderately distributed in breast tumor samples (Figure 7).

**Figure 8:**
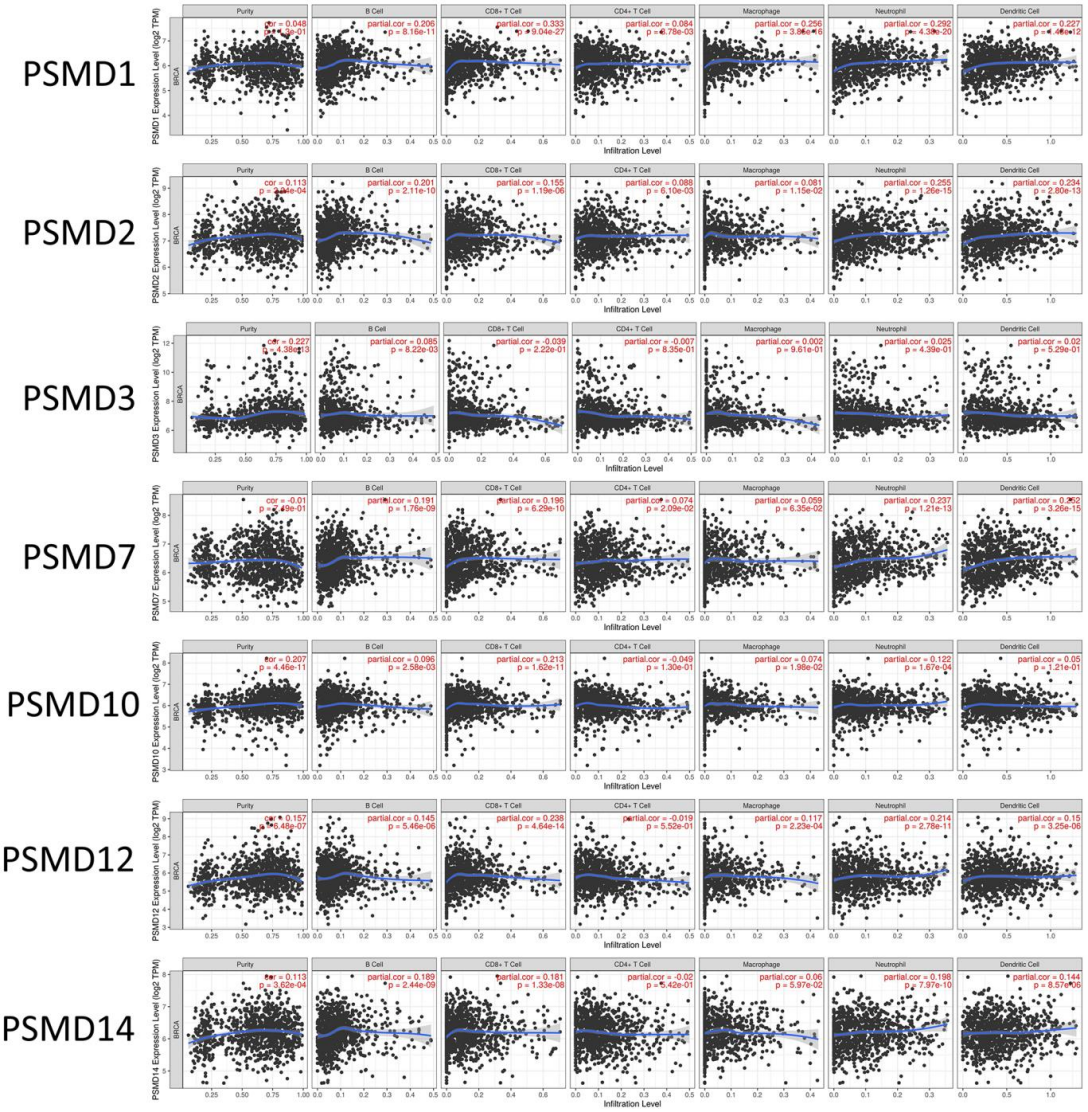
**Correlations between expressions of 26S proteasome delta subunit, non-ATPase (PSMD) family members and immune infiltration profiles of breast cancer via the TIMER database.** The figure shows correlations between each abnormally expressed gene of the *PSMD* family and levels of several tumor-infiltrating immune cell markers, such as B cells, cluster of differentiation 8-positive (CD8^+^) T cells, CD4^+^ T cells, macrophages, neutrophils, and dendritic cells.

In addition, when we performed the required analysis using the Tumor Immune Estimation Resource (TIMER) database (available at: http://timer.cistrome.org/), *PSMD* member genes also showed relevance to immune infiltration profiles of BRCA, and the expression of each individual was related to tumor purity and markers of six tumor-infiltrating immune cell types which belonged to two separate groups: a lymphoid lineage (B cells, cluster of differentiation 4-positive (CD4^+^) T cells, and cluster of differentiation 8-positivie (CD8+) T cells) and myeloid lineage (neutrophils, macrophages, and dendritic cells) (Figure 8).

### Pathway and network analysis of *PSMD* family genes

**Figure 9:**
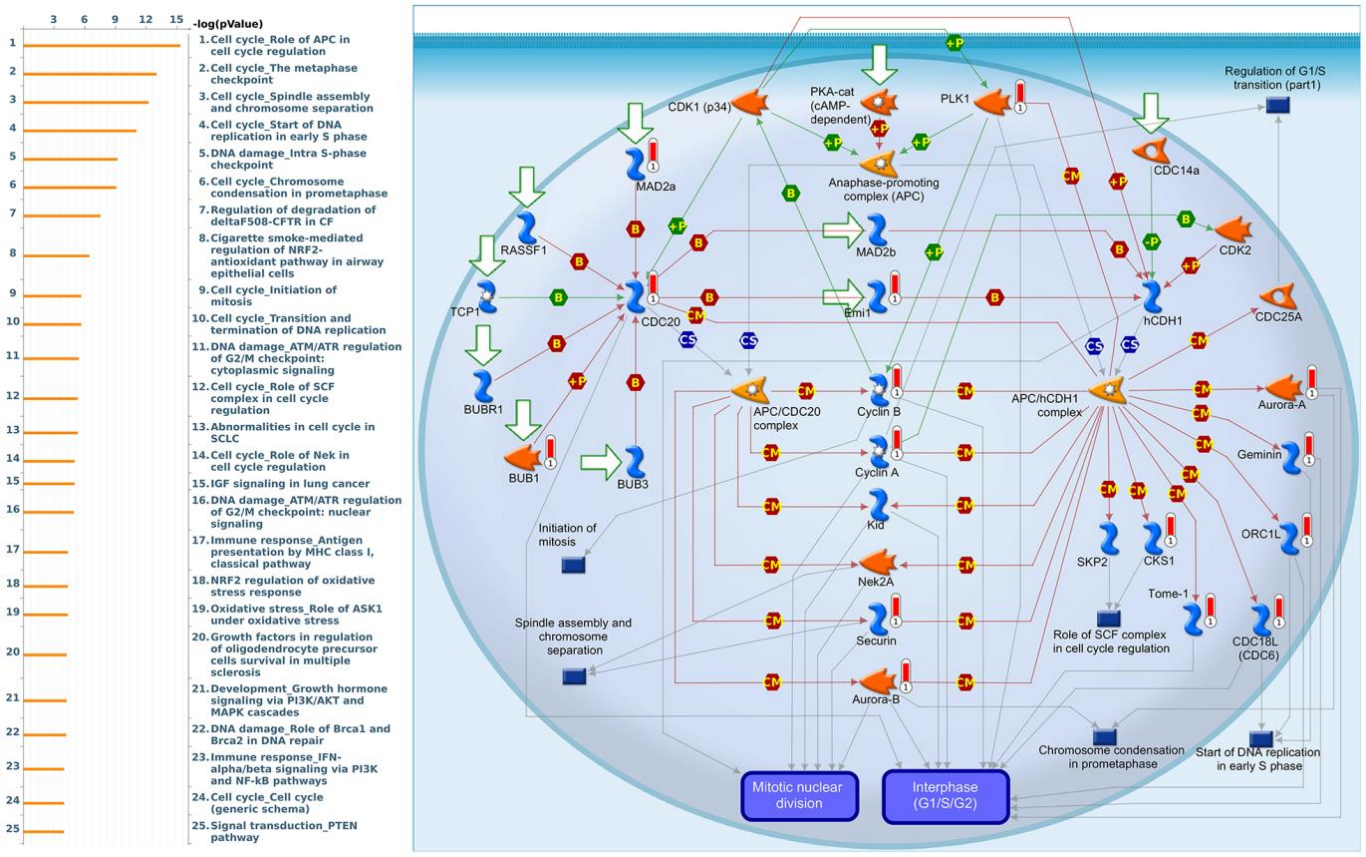
**Cell cycle-related networks correlated with the 26S proteasome delta subunit, non-ATPase 1 (*PSMD1*) family gene in breast cancer (BRCA).** MetaCore pathway analysis of biological processes revealed that pathways related to "Cell cycle_Role of APC in cell cycle regulation" were correlated with BRCA development.

**Figure 10:**
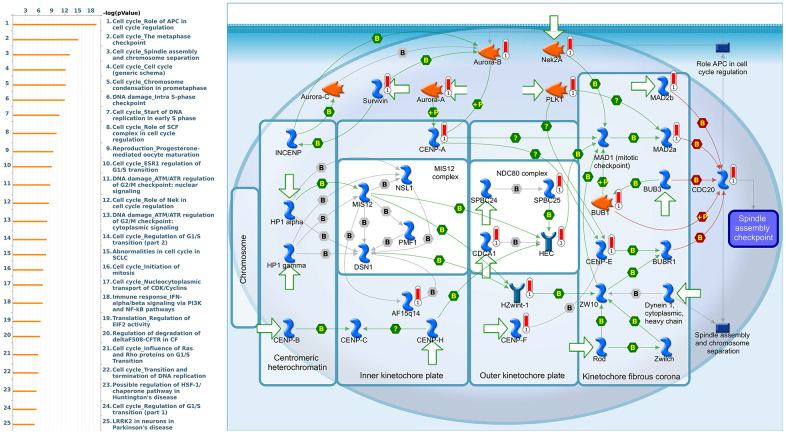
**Cell cycle-related networks correlated with the 26S proteasome delta subunit, non-ATPase 2 (*PSMD2*) family gene in breast cancer (BRCA).** MetaCore pathway analysis of biological processes revealed that pathways related to "Cell cycle_The metaphase checkpoint" were significantly associated with BRCA development.

**Figure 11:**
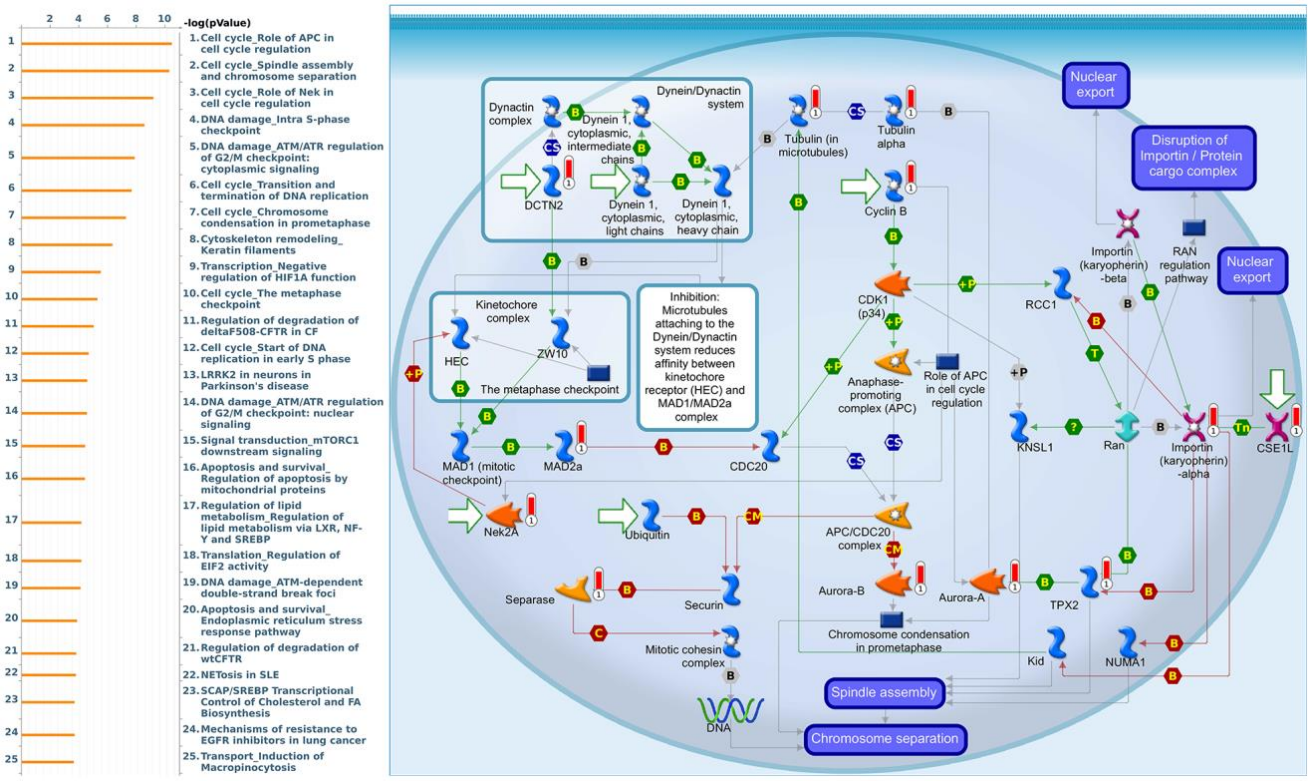
**Cell cycle-related networks correlated with the 26S proteasome delta subunit, non-ATPase 3 (*PSMD3*) family gene in breast cancer (BRCA).** MetaCore pathway analysis of biological processes revealed that pathways related to "Cell cycle_Spindle assembly and chromosome separation" were significantly associated with BRCA development.

**Figure 12:**
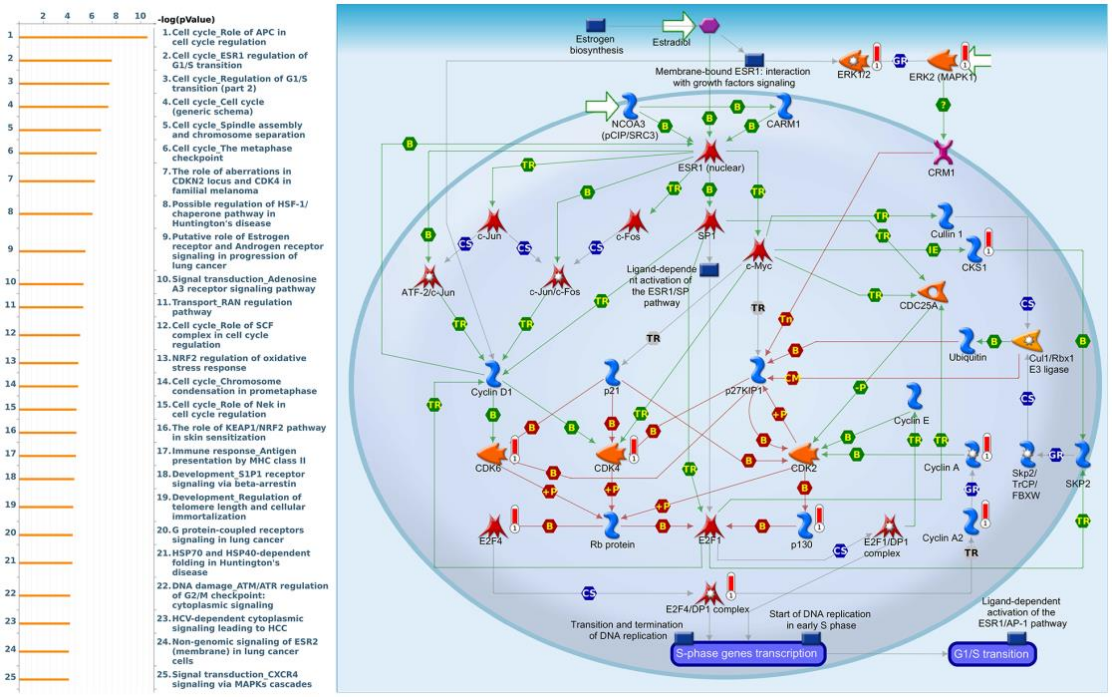
**Cell cycle-related networks correlated with the 26S proteasome delta subunit, non-ATPase 7 (*PSMD7*) family gene in breast cancer (BRCA).** MetaCore pathway analysis of biological processes revealed that pathways related to "Cell cycle_ESR1 regulation of G1S transition" were significantly associated with BRCA development.

**Figure 13:**
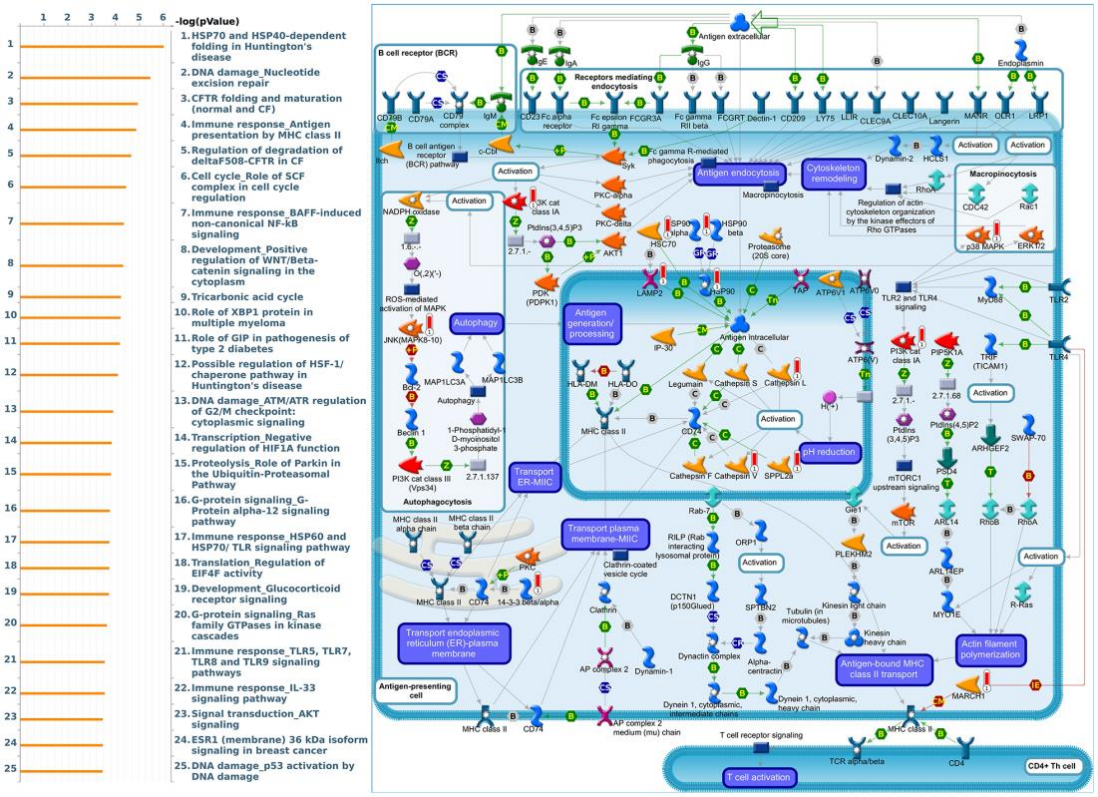
**Cell cycle-related networks correlated with the 26S proteasome delta subunit, non-ATPase 10 (*PSMD10*) family gene in breast cancer (BRCA).** MetaCore pathway analysis of biological processes revealed that pathways related to "Immune response_Antigen presentation by MHC class II" were significantly associated with BRCA development.

**Figure 14:**
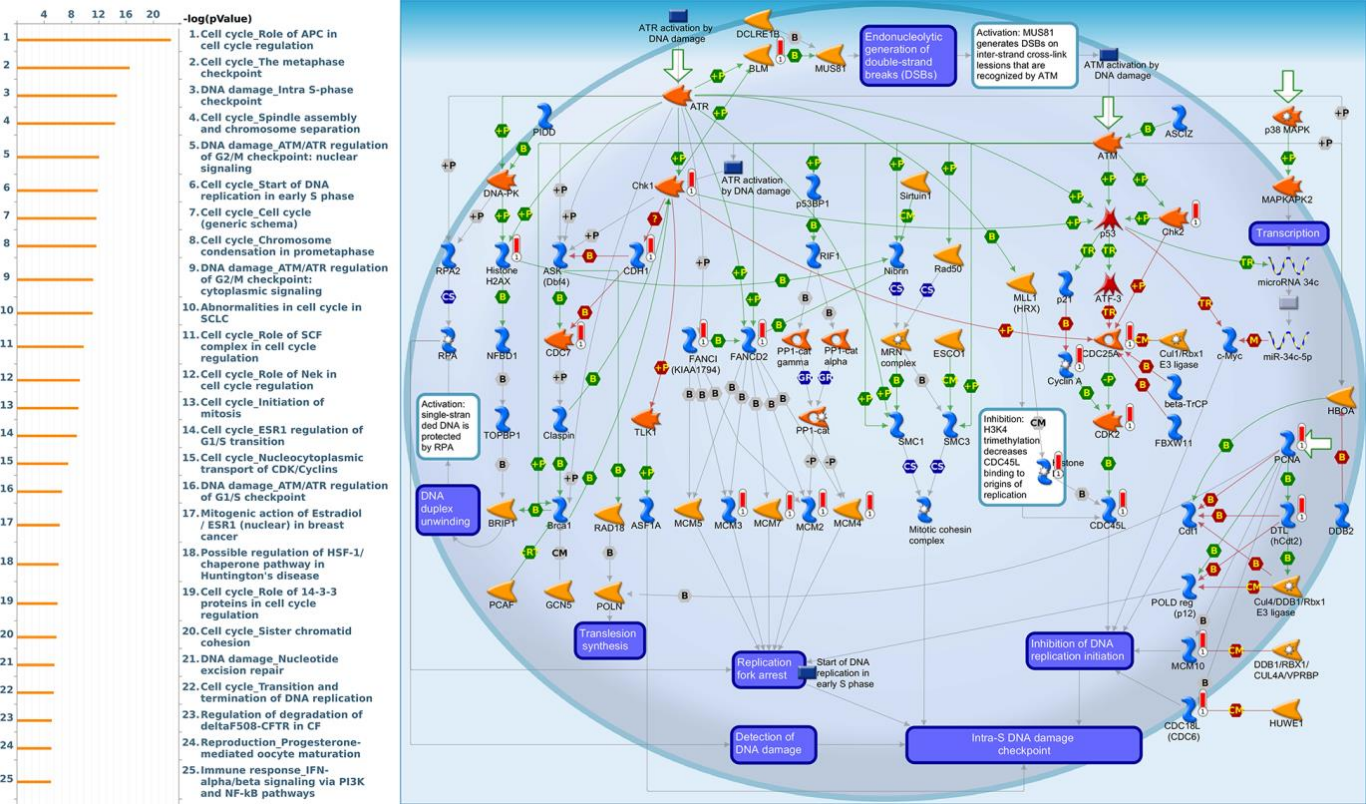
**Cell cycle-related networks correlated with the 26S proteasome delta subunit, non-ATPase 12 (*PSMD12*) family gene in breast cancer (BRCA).** MetaCore pathway analysis of biological processes revealed that pathways related to "DNA damage_Intra S-phase checkpoint" were significantly associated with BRCA development.

**Figure 15:**
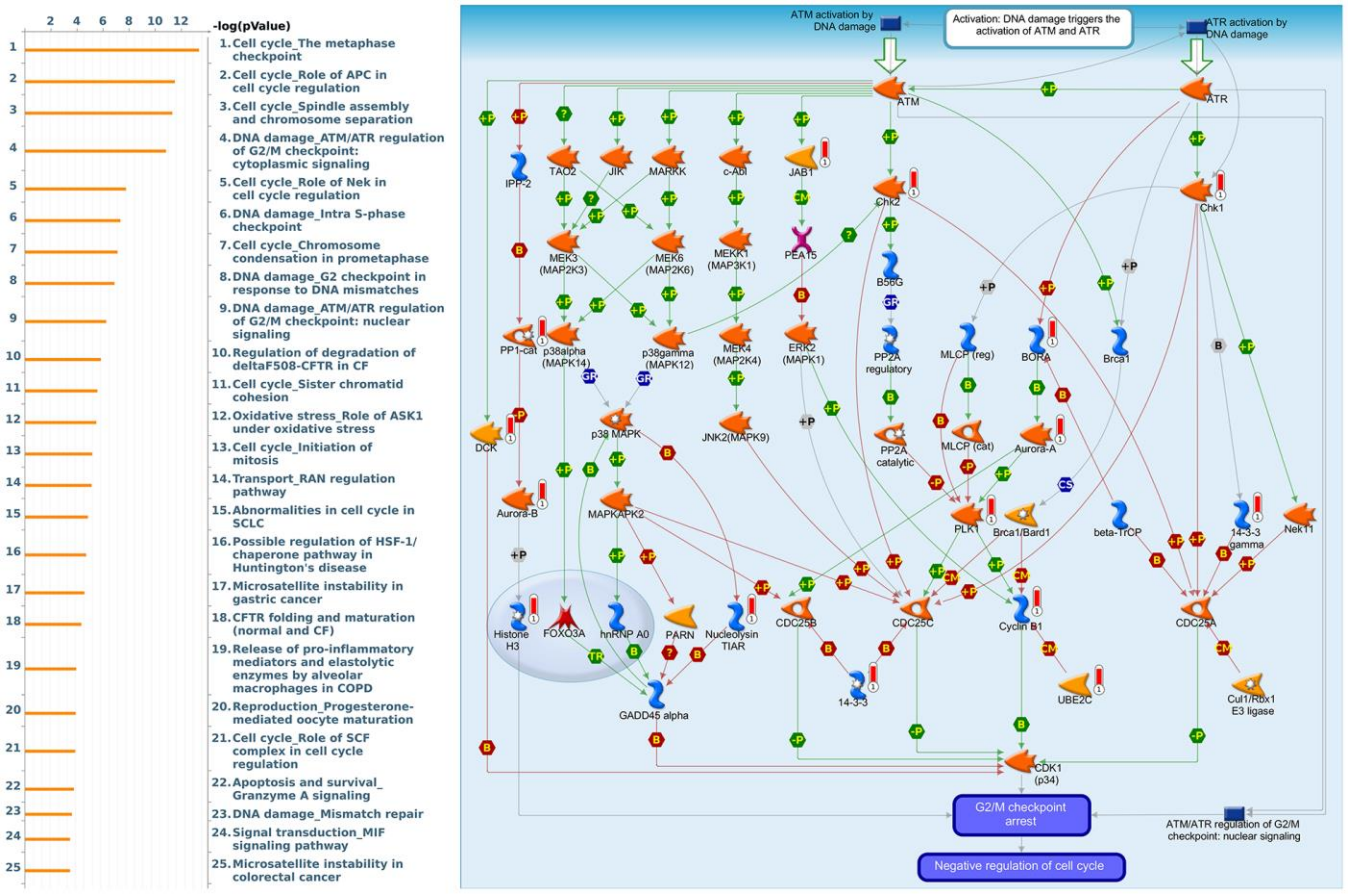
**Cell cycle-related networks correlated with the 26S proteasome delta subunit, non-ATPase 14 (*PSMD14*) family gene in breast cancer (BRCA).** MetaCore pathway analysis of biological processes revealed that pathways related to "DNA damage_ATMATR regulation of G_2_M checkpoint cytoplasmic signaling" were significantly associated with BRCA development.

Since some potential information for refining the full picture of regulated pathways available to *PSMD* family genes is still missing, GeneGo Metacore software was launched to extensively explore downstream networks linked to the aforementioned co-expression patterns of *PSMD* family genes. We obtained PSMD1 coexpression profiles of BRCA from available datasets from both METABRIC and TCGA. As a result, annotations of biological processes obtained from GeneGo Metacore showed that genes co-expressed with PSMD1 participated in several networks and cell cycle-related pathways such as “Cell cycle_Role of APC in cell cycle regulation”, “Cell cycle_The metaphase checkpoint”, “Cell cycle_Spindle assembly and chromosome separation”, “DNA damage_Intra S-phase checkpoint”, and “Cell cycle_Start of DNA replication in early S phase” (Figure 9 and Supplementary Table 2). PSMD2 was associated with “Cell cycle_Cell cycle (generic schema) Cell cycle_Start of DNA replication in early S phase”, “Cell cycle_Chromosome condensation in prometaphase”, “DNA damage_Intra S-phase checkpoint”, “Cell cycle_Role of SCF complex in cell cycle regulation”, and “Reproduction_Progesterone-mediated oocyte maturation” (Figure 10 and Supplementary Table 3). PSMD3 was involved in “Cell cycle_Role of Nek in cell cycle regulation”, “Transcription_Negative regulation of HIF1A function”, “DNA damage_Intra S-phase checkpoint”, “DNA damage_ATM/ATR regulation of G2/M checkpoint: cytoplasmic signaling”, “Cytoskeleton remodeling_Keratin filaments”, and “Regulation of degradation of deltaF508-CFTR in CF” (Figure 11 and Supplementary Table 4). PSMD7 was involved in “Cell cycle_ESR1 regulation of G1/S transition”, “The role of aberrations in CDKN2 locus and CDK4 in familial melanoma”, “Putative role of estrogen receptor and androgen receptor signaling in the progression of lung cancer”, “Signal transduction_Adenosine A3 receptor signaling pathway”, and “Transport_RAN regulation pathway” (Figure 12 and Supplementary Table 5). PSMD10 was involved in “DNA damage_Nucleotide excision repair”, “CFTR folding and maturation (normal and CF)”, “Immune response_Antigen presentation by MHC class II”, “Regulation of degradation of deltaF508-CFTR in CF”, “Cell cycle_Role of SCF complex in cell cycle regulation”, and “Immune response_BAFF-induced non-canonical NF-kB signaling” (Figure 13 and Supplementary Table 6). PSMD12 was involved in “DNA damage_ATM/ATR regulation of G_2_/M checkpoint: nuclear signaling”, “Cell cycle_Initiation of mitosis”, “Cell cycle_ESR1 regulation of G_1_/S transition”, “Cell cycle_Nucleocytoplasmic transport of CDK/cyclins”, and “Mitogenic action of estradiol/ESR1 (nuclear) in breast cancer” (Figure 14 and Supplementary Table 7). PSMD14 was involved in “Cell cycle_The metaphase checkpoint”, “Regulation of degradation of deltaF508-CFTR in CF”, “Cell cycle_Sister chromatid cohesion”, “Oxidative stress_Role of ASK1 under oxidative stress”, and “Transport_RAN regulation pathway” (Figure 15 and Supplementary Table 8). Meanwhile, we obtained similar results from the cBioPortal and the Cytoscape and METABRIC databases, which revealed that these PSMD members were correlated with metabolic pathways and the cancer development-related genes (Supplementary Figure 2).

## DISCUSSION

Recent epidemiologic studies indicated that BRCA has been displaced lung cancer in term of the most frequently diagnosed cases among women globally. Despite some improvements having been made in medical and surgical treatments of BRCA, a shortage of detection methods for early screening or diagnosis, accompanied by high risks of metastasis, chemoresistance, endocrine-resistance, and recurrence has resulted in a top ranking in overall mortality for this disease, which still needs to be fully investigated. Therefore, identifying specific key molecular pathways and highly sensitive, reliable biomarkers is urgently needed [48–53]. In recent times, the rapid growth of microarray and high-throughput sequencing data has provided convenient and comprehensive online platforms to elucidate the pathogenesis of tumors, which has allowed us to properly monitor tumor progression and prognoses [22–26].

Based on the results of this study, it suggested that most of the PSMD family are generally dysregulated in hundreds of distinctive types of cancers. On the other hand, expression profiles indicated that this family's genes not only accompany tumor multi-stage progression but are also involved in other tumor-related issues. For instance, upregulation of the PSMD1 gene was mainly enriched alongside a rise in tamoxifen resistance displayed by BRCA cells [55]. The autophagic degradation of 19S proteasomal subunits of both PSMD1 and PSMD2 were mediated by ATG16 [56]. PSMD3 is believed to be involved in stabilizing HER2, a growth-promoting protein on the exterior of all breast cells, from degradation [57]. Upregulation of the PSMD4 gene by hypoxic conditions in prostate cancer cells suggests a novel therapy for treatment [58]. PSMD7 was significantly linked to earlier stimulation of prostate cancer [59]. PSMD10 overexpression was supposed to substantially contribute to the onset of tumors as observed in various cancer types [60]. PSMD11 is a novel biomarker of pancreatic cancer progression [61]. High levels of PSMD12 enhanced both the proliferation and invasion of BRCA and gliomas, one of the fastest-growing and most aggressive brain neoplasms, by upregulating nuclear factor erythroid 2-related factor 2 (Nrf2) [62]. In the case of proteasomal degradation, consistently high levels of PSMD14, which regulates the de-ubiquitination substrate, may lead to a worse prognosis of lung adenocarcinomas [63]. The recent literature indicated that PSMDs play important roles in various cancers, and may represent possible biomarkers for predicting clinical out-comes and precise diagnoses, which provides promising molecular targets for the research and development of drugs and targeted therapies.

Despite extensive efforts having been made to properly understand the roles of each PSMD family member in various clinical diseases and cancer development, there is still limited evidence regarding relationships between all PSMD family genes and BRCA. We therefore conducted this study using available public databases to analyze possible biological regulation of PSMD family genes along with the occurrence and the development of BRCA. The data revealed that higher mRNA and protein levels of PSMD1, PSMD2, PSMD3, PSMD7, PSMD10, PSMD12, and PSMD14 lead to worse prognoses in terms of both DMFS and RFS. Therefore, we chose these PSMD family genes for further bioinformatics analyses. Moreover, the coexpression and pathway analysis also revealed the involvement of these family genes together with cell metabolism, immune responses, cyclin-dependent kinases (CDKs), and other cell-cycle pathways and signaling networks. The current study was consistent with the previous literature; these results credibly suggest that some specific genes of the PSMD family act as oncogenes, whose differential expressions may serve as potential molecular biomarkers in terms of diagnosis, classification, and prognosis for developing BRCA treatments.

Based on our knowledge, this is the first ever report on PSMD family genes expression in relation to patient survival prediction in BRCA. Most of all, since various types of high-throughput databases were integrated and some underlying biological mechanism were revealed that PSMD genes show prognostic and predictive value in BRCA, hence they may possibly serve as novel biomarkers in malignancy screening and/or potential prognosticators in assessing BRCA severity and prognosis.

## MATERIALS AND METHODS

### Oncomine and UALCAN analysis

Oncomine, available at (https://www.oncomine.org), is generally recognized as a bioinformatics analytical tool for gene expression microarrays among PSMD family members [64]. Differences in expression between normal tissues and 20 types of cancer counterparts were comprehensively evaluated, under conditions that thresholds of three parameters were adjusted to a multiple of change >2; p<0.0001; and gene ranked in the top 10%; with data type as “all”. Numbers of significant unique analyses that met the selection criteria in BRCA are presented as digits, while overexpressed and under-expressed genes are displayed in red and blue gradients, respectively, in descending order of the gene rank percentile. In the subsequent stage, the ggpubr package in R environment was run to obtain plots of BRCA subtypes as we previously described [65–68].

Transcriptomic expressions of PSMD family members were analyzed in BRCA sample using the UALCAN (http://ualcan.path.uab.edu/) platform. UALCAN collected TCGA level 3 RNA-Seq and clinical data from different cancer types. With genes of interest, UALCAN allows users to perform biomarkers identification to verify gene expressions with multiple clinical factors. A boxplot was drawn of PSMD mRNA expression levels measured in BRCA specimens (red) compared to their normal counterparts (blue) obtained from the UALCAN database. Statistical analysis was performed using Student’s t-test, and p<0.05 was considered statistically significant [69].

### Evaluation of differential PSMD expressions in cancer cell lines by a cancer cell line encyclopedia (CCLE) analysis

To further search for individual expression levels of *PSMD* family genes on a larger scale, the CCLE project (available at https://portals.broadinstitute.org/ccle) was launched [70]. 1000 This web-based tool offers public access to both genetic and pharmacologic characterizations of numerous human cancer models, including over human cancer cell lines and over 130,000 unique datasets. Moreover, the integrated RNA-Seq Aligned Reads tool was applied to 60 independent BRCA cell lines prior to plotting expressions of *PSMD* family members one at a time [71–73].

### Kaplan-Meier (KM) overall survival analysis

The KM database (https://kmplot.com/), an integrated online database well-known for assessing target genes of survivors among 21 cancer types, was subsequently leveraged to further expand some prognosis-related issues. By concurrently integrating mRNA expression levels and clinical data obtained from target genes, the independent prognostic values of PSMD target genes on patients diagnosed with BRCA, including both distant metastasis-free survival (DMFS) and relapse-free survival (RFS), were represented as KM survival plots of two distinct groups of patients. Comparisons of the two patient cohorts were performed with 95% confidence intervals of hazard ratios (HRs) and fixed log-rank *p* values [74].

### Analysis of protein expressions in clinical human specimens

The Human Protein Atlas (HPA, https://www.proteinatlas.org) provides a wealth of information on sequences, pathology, expressions, and distributions in various cancer tissues. The first version of this database contained more than 400,000 high-resolution images corresponding to more than 700 antibodies to human proteins [75]. This study analyzed the differential status of protein expressions and the localization of select PSMD family protein expression in breast tissue.

### Functional enrichment analysis of *PSMD* target genes

To visualize genomics datasets on a large scale, particularly TCGA and METABRIC databases (available at the cBioPortal platform), the InteractiVenn tool (http://www.interactivenn.net/) was chosen to draw a one-way Venn diagram which illustrates the overlap and numbers of genes associated with expressions of *PSMD* target genes across the two given datasets [76]. The intersection between the two sets was subsequently analyzed for related pathways and involved networks using the online MetaCore platform (https://portal.genego.com/), with p-value of <0.05, as we previously described [77–82].

### Tumor immune estimation resource (TIMER) database analysis

TIMER vers. 2.0 (available at http://timer.comp-genomics.org/) is generally known as a trustworthy resource for systematic analysis of host immune infiltrates across multiple cancer types and related diseases. In other words, this webserver can help estimate abundances of six given immune cell types which belong to two separate groups: the lymphoid lineage (B cells, cluster of differentiation 4-positivie (CD4^+^) T cells, and cluster of differentiation 8-positivie (CD8^+^) T cells) and myeloid lineage (neutrophils, macrophages, and dendritic cells) in the tumor microenvironment, under the DiffExp module with default parameters. Finally, correlations were illustrated as a scatterplot, while *PSMD* gene expression levels were represented on the x-axis and related tumor-infiltrating immune cell markers were represented on the y-axis [83, 84].

## Supplementary Material

Supplementary Figures

Supplementary Tables
