# Supplementary material for: Prognostic and immune infiltration signatures of proteasome 26S subunit, non-ATPase (PSMD) family genes in breast cancer patients
Source: Aging (Albany NY). 2021 Nov 28;13(22):24882–913. doi: 10.18632/aging.203722 (PMC8660617; doi:10.18632/aging.203722)
Supplement: Supplementary Figures [file aging-13-203722-s001.pdf]

SUPPLEMENTARY FIGURES

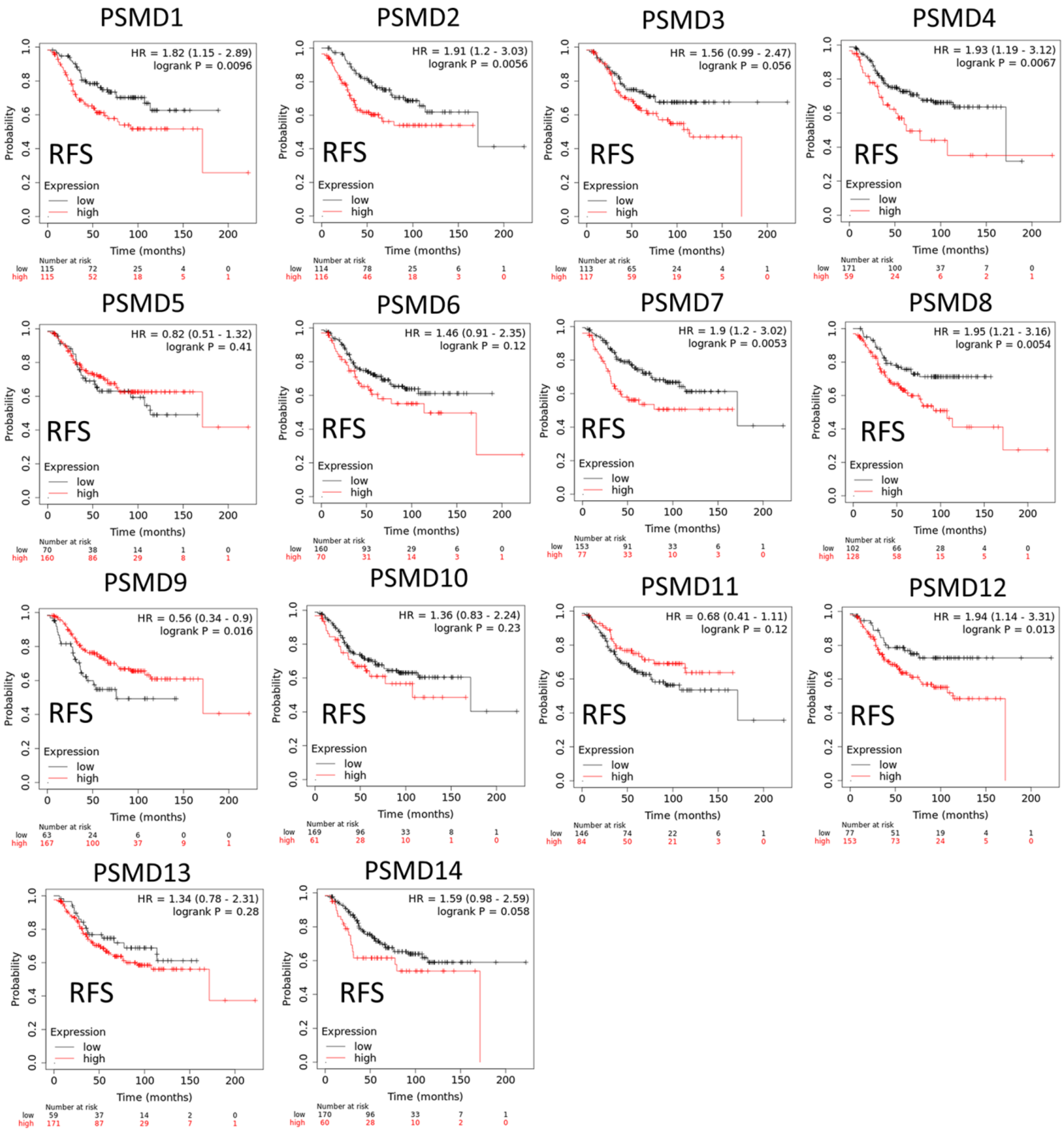

Supplementary Figure 1. Prognostic values of 26S proteasome delta subunit, non-ATPase (PSDM) family genes in breast cancer (BRCA) patients (GSE21653 database). A recurrence metastasis-free sur-vival (RFS) dataset was used for the analysis. An auto-cutoff strategy was set in this analysis to differentiate patients into two groups based on the value of PSMDs mRNAs. The two survival curves respectively illustrate survival outcomes (including survival per-centages and survival times) of BRCA patients with high (red) or low (black) expression levels of PSMD family members. Increased mRNA levels of most PSMD family genes resulted in poor prognoses, while an increasing level of PSMD9 was associated with favorable outcomes (p<0.05 was considered statistically significant).

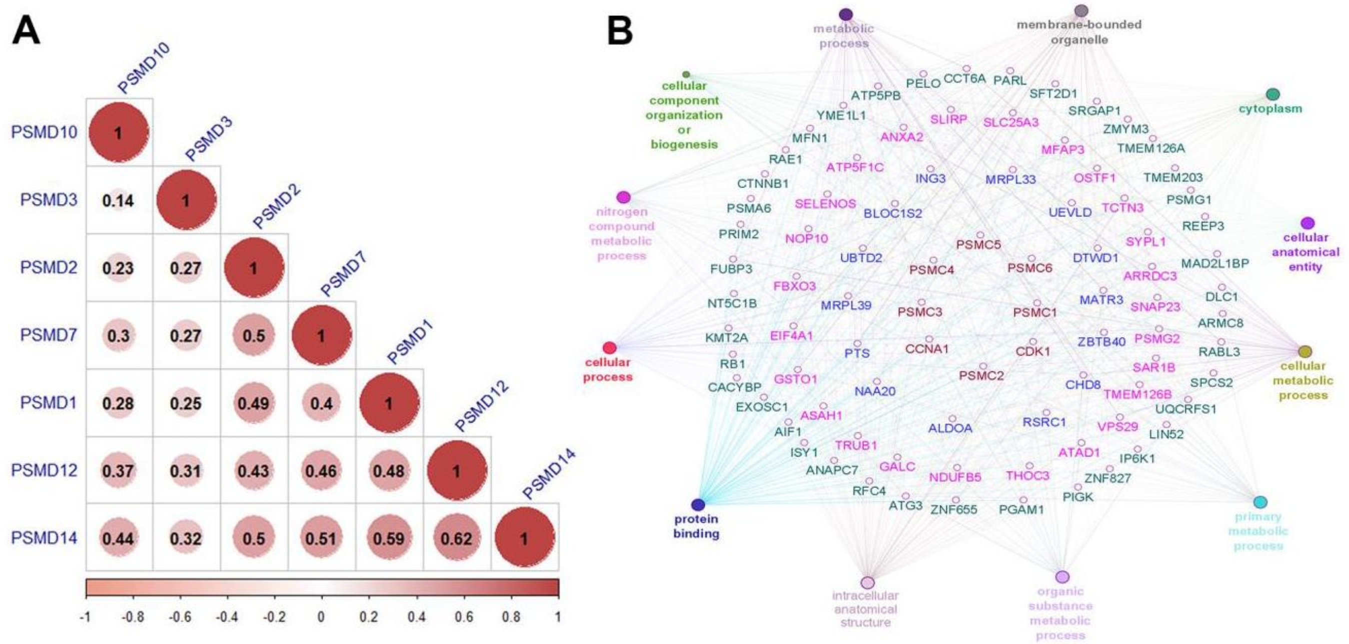

Supplementary Figure 2. Correlations among different 26S proteasome delta subunit, non-ATPase (PSMD) family members in breast cancer (BRCA). (**A**) Correlations between PSMD family members and cell-cycle-related genes in BRCA patients from the METABRIC database, and in-significant correlations are marked by crosses. (**B**) Through a Cytoscape analysis, high correlations between PSMD members and cancer development-related pathways were observed.
