# Supplementary material for: Prognostic and immune infiltration signatures of proteasome 26S subunit, non-ATPase (PSMD) family genes in breast cancer patients
Source: Aging (Albany NY). 2021 Nov 28;13(22):24882–913. doi: 10.18632/aging.203722 (PMC8660617; doi:10.18632/aging.203722)
Supplement: Supplementary Tables [file aging-13-203722-s002.pdf]

## SUPPLEMENTARY TABLES

**Supplementary Table 1. Univariate and multivariate Cox proportional hazards regression analysis of breast cancer (BRCA) overall survival (OS) outcomes.**

| Variables | Patient number | Univariate | | Multivariate | |
|---|---|---|---|---|---|
| | | HR (95% CI) | p value | HR (95% CI) | p value |
| **Age (year)** | | | | | |
| < 60 | 533 | reference | 0.0001 | reference | 0.000165 *** |
| > 60 | 461 | 1.97 (1.40 – 2.77) | | 1.966 (1.383 – 2.795) | |
| **Gender** | | | | | |
| Male | 11 | reference | 0.956 | | |
| Female | 983 | 0.945 (0.132 – 6.78) | | | |
| **Tumor stage** | | | | | |
| Stage I/II | 740 | reference | | reference | |
| Stage III/IV | 236 | 2.791 (1.96 – 3.97) | 1.2e-08 *** | 3.4 (1.825 – 6.34) | 0.000116 *** |
| Stage X | 18 | 2.56 (1.17 – 5.6) | 0.0189 * | 3.503 (0.97 – 12.61) | 0.055078 |
| **T** | | | | | |
| T1/T2 | 841 | reference | | reference | |
| T3/T4 | 150 | 1.85 (1.25 – 2.73) | 0.0019* | 0.834 (0.494 – 1.4) | 0.496934 |
| TX | 3 | 0.527 (0.072 – 3.84) | 0.527 | 0.098 (0.01 – 0.89) | 0.039511 * |
| **N** | | | | | |
| N0/N1 | 799 | reference | | reference | |
| N2/N3 | 176 | 2.32 (1.547 – 3.484) | 4.75e-05 *** | 0.784 (0.44 – 1.39) | 0.407585 |
| NX | 19 | 3.97 (2.06 – 7.65) | 3.73e-05 *** | 2.79 (1.154 – 6.76) | 0.022777 * |
| **M** | | | | | |
| M0 | 834 | reference | | reference | |
| M1 | 20 | 5.296 (3.09 – 9.05) | 1.08e-09 *** | 1.2 (0.5 – 2.6) | 0.62 |
| MX | 140 | 1.396 (0.778 – 2.5) | 0.262 | | |
| **PSMD1 expression** | | | | | |
| Low | 497 | reference | 0.064 | | |
| High | 497 | 1.4 (0.98 – 2) | | | |
| **PSMD2 expression** | | | | | |
| Low | 497 | reference | 0.457 | | |
| High | 497 | 1.137 (0.81 – 1.59) | | | |
| **PSMD3 expression** | | | | | |
| Low | 497 | reference | 0.421 | | |
| High | 497 | 1.149 (0.81 – 1.61) | | | |
| **PSMD7 expression** | | | | | |
| Low | 497 | reference | 0.343 | | |
| High | 497 | 1.178 (0.84 – 1.7) | | | |
| **PSMD10 expression** | | | | | |
| Low | 497 | reference | 0.0035 ** | reference | 0.001508 ** |
| High | 497 | 1.68 (1.188 – 2.396) | | 1.798 (1.251 – 2.585) | |
| **PSMD12 expression** | | | | | |
| Low | 497 | reference | 0.168 | | |
| High | 497 | 1.27 (0.9 – 1.792) | | | |
| **PSMD14 expression** | | | | | |
| Low | 497 | reference | 0.127 | | |
| High | 497 | 1.3 (0.93 – 1.836) | | | |

Factors showing significant relationships with OS from a univariate analysis were then used for a multi-variate analysis. HR, hazard ratio; CI, confidence interval; * p<0.05.

**Supplementary Table 2. Pathway analysis of genes co-expressed with 26S proteasome delta subunit, non-ATPase 1 (PSMD1) from public breast cancer (BRCA) databases using the MetaCore platform (with p<0.01 set as the cutoff value).**

| No. | Map | *p*-Value | Network objects from active data |
|---|---|---|---|
| 1 | Cell cycle_Role of APC in cell cycle regulation | 4.58E-16 | BUB1, CDC18L (CDC6), Tome-1, Geminin, Emi1, Cyclin A, Aurora-A, PLK1, Aurora-B, CDC20, Cyclin B, MAD2a, Securin, ORC1L, CKS1 |
| 2 | Cell cycle_The metaphase checkpoint | 1.10E-13 | BUB1, SPBC25, CENP-A, Aurora-A, PLK1, Aurora-B, HEC, CDC20, HZwint-1, CENP-F, MAD2a, Survivin, CENP-E, AF15q14 |
| 3 | Cell cycle_Spindle assembly and chromosome separation | 6.96E-13 | Importin (karyopherin)-alpha, TPX2, CSE1L, Aurora-A, KNSL1, Aurora-B, HEC, CDC20, Tubulin alpha, Cyclin B, MAD2a, Separase, Securin |
| 4 | Cell cycle_Start of DNA replication in early S phase | 1.10E-11 | CDC18L (CDC6), Geminin, DP1, MCM4, MCM3, Cyclin E, MCM10, ORC6L, MCM4/6/7 complex, MCM2, ORC1L, CDC45L |
| 5 | DNA damage_Intra S-phase checkpoint | 8.17E-10 | PCNA, CDC18L (CDC6), BLM, FANCD2, DTL (hCdt2), Histone H2AX, MCM4, MCM3, Cyclin A, Chk1, MCM7, MCM10, MCM2, Histone H3, CDC45L |
| 6 | Cell cycle_Chromosome condensation in prometaphase | 1.08E-09 | CAP-C, Cyclin A, CAP-G/G2, Aurora-A, Aurora-B, CAP-E, Cyclin B, TOP2, Histone H3 |
| 7 | Regulation of degradation of deltaF508-CFTR in CF | 3.98E-08 | Csp, HSP70, RNF4, UFD1, SUMO-2, Derlin1, UCHL1, Hdj-2, SUMO-3, HSC70 |
| 8 | Cigarette smoke-mediated regulation of NRF2-antioxidant pathway in airway epithelial cells | 5.08E-07 | PRDX1, TXNRD1, NRF2, SRX1, GCL reg, ME1, TALDO, DJ-1 |
| 9 | Cell cycle_Initiation of mitosis | 3.22E-06 | Nucleolin, PLK1, KNSL1, Cyclin B2, FOXM1, Kinase MYT1, Histone H3 |
| 10 | Cell cycle_Transition and termination of DNA replication | 3.22E-06 | TOP2 alpha, PCNA, Bard1, Cyclin A, MCM2, TOP2, FEN1 |
| 11 | DNA damage_ATM/ATR regulation of G2/M checkpoint: cytoplasmic signaling | 5.62E-06 | UBE2C, JAB1, Chk1, Aurora-A, PLK1, Aurora-B, DCK, Histone H3, 14-3-3 |
| 12 | Cell cycle_Role of SCF complex in cell cycle regulation | 7.15E-06 | Emi1, Cyclin E, Chk1, PLK1, RING-box protein 1, NEDD8, CKS1 |
| 13 | Abnormalities in cell cycle in SCLC | 7.15E-06 | PCNA, Cyclin A, Cyclin E, Aurora-B, Histone H3, Cyclin E2, CKS1 |
| 14 | Cell cycle_Role of Nek in cell cycle regulation | 1.44E-05 | TPX2, Aurora-A, PI3K cat class IA, HEC, Tubulin alpha, MAD2a, Histone H3 |
| 15 | IGF signaling in lung cancer | 1.49E-05 | 4E-BP1, Histone H2AX, PI3K cat class IA, SOS, RHEB2, Survivin, mTOR, GRB2 |
| 16 | DNA damage_ATM/ATR regulation of G2/M checkpoint: nuclear signaling | 1.78E-05 | CDC18L (CDC6), Histone H2AX, Cyclin A, Chk1, PLK1, Cyclin B, Cyclin B2, TTK |
| 17 | Immune response_Antigen presentation by MHC class I, classical pathway | 7.00E-05 | PSMB5, HSP70, TAP1 (PSF1), IDE, Nardilysin, TAP, PSMB2, TAP2 (PSF2) |
| 18 | NRF2 regulation of oxidative stress response | 7.00E-05 | Thioredoxin, PRDX1, TXNRD1, NRF2, GCL reg, PI3K cat class IA, SOD1, DJ-1 |
| 19 | Oxidative stress_Role of ASK1 under oxidative stress | 7.00E-05 | HPK38, UNRIP, Thioredoxin, PRDX1, MT-TRX, 14-3-3 zeta/delta, SOD1, 14-3-3 |
| 20 | Growth factors in regulation of oligodendrocyte precursor cells survival in multiple sclerosis | 9.25E-05 | 4E-BP1, 14-3-3 beta/alpha, CD80, PI3K cat class IA, 14-3-3 zeta/delta, Caspase-3, mTOR |
| 21 | Development_Growth hormone signaling via PI3K/AKT and MAPK cascades | 9.25E-05 | 4E-BP1, ATF-2, Elk-4, SOS, RHEB2, mTOR, GRB2 |
| 22 | DNA damage_Role of Brca1 and Brca2 in DNA repair | 1.03E-04 | PCNA, FANCD2, Histone H2AX, Rad51, MSH6, Bard1 |
| 23 | Immune response_IFN-alpha/beta signaling via PI3K and NF-κB pathways | 1.60E-04 | PCNA, 4E-BP1, Cyclin A, Cyclin E, GBP1, p19, PI3K cat class IA, DHFR, RSAD2, ISG15 |
| 24 | Cell cycle_Cell cycle (generic schema) | 1.66E-04 | E2F5, DP1, Cyclin A, Cyclin E, Cyclin B |
| 25 | Signal transduction_PTEN pathway | 1.68E-04 | PCNA, PI3K cat class IA, SOS, Caspase-3, RHEB2, mTOR, GRB2 |

**Supplementary Table 3. Pathway analysis of genes co-expressed with 26S proteasome delta subunit, non-ATPase 2 (PSMD2) from public breast cancer databases using the MetaCore platform (with p<0.01 set as the cutoff value).**

| No. | Map | *p*-Value | Network objects from active data |
|---|---|---|---|
| 1 | Cell cycle_Role of APC in cell cycle regulation | 5.61E-20 | Nek2A, BUB1, MAD2b, CDC18L (CDC6), Tome-1, Emi1, Cyclin A, Aurora-A, PLK1, Aurora-B, CDC25A, CDC20, SKP2, Cyclin B, MAD2a, Securin, ORC1L, CDK2, CKS1 |
| 2 | Cell cycle_The metaphase checkpoint | 9.51E-16 | Nek2A, BUB1, MAD2b, SPBC25, CENP-A, Aurora-A, PLK1, Aurora-B, HEC, CDCA1, CDC20, HZwint-1, CENP-F, MAD2a, Survivin, CENP-E, AF15q14 |
| 3 | Cell cycle_Spindle assembly and chromosome separation | 9.84E-14 | Nek2A, Importin (karyopherin)-alpha, TPX2, CSE1L, Aurora-A, KNSL1, Aurora-B, HEC, CDC20, Tubulin alpha, Cyclin B, MAD2a, Separase, Securin, Tubulin (in microtubules) |
| 4 | Cell cycle_Cell cycle (generic schema) | 8.83E-13 | CDC25C, CDK4, DP1, p107, Cyclin A, Cyclin E, CDC25A, Cyclin B, E2F2, CDC25B, E2F4, CDK2 |
| 5 | Cell cycle_Chromosome condensation in prometaphase | 8.83E-13 | CAP-H/H2, Condensin, CAP-C, Cyclin A, CNAP1, CAP-G/G2, Aurora-A, CAP-D2/D3, Aurora-B, CAP-E, Cyclin B, TOP2 |
| 6 | DNA damage_Intra S-phase checkpoint | 1.45E-12 | TOPBP1, CDC18L (CDC6), BLM, FANCD2, DTL (hCdt2), Chk2, MCM4, MCM3, Cyclin A, Chk1, FANCI (KIAA1794), PP1-cat, CDC25A, MCM7, MCM10, PP1-cat alpha, CDC7, MCM2, CDK2, CDC45L |
| 7 | Cell cycle_Start of DNA replication in early S phase | 2.63E-11 | CDC18L (CDC6), DP1, MCM4, MCM3, Cyclin E, MCM10, ORC6L, MCM4/6/7 complex, CDC7, MCM2, ORC1L, CDK2, CDC45L |
| 8 | Cell cycle_Role of SCF complex in cell cycle regulation | 1.2E-10 | Cullin 1, CDK4, Emi1, Cyclin E, Skp2/TrCP/FBXW, Chk1, PLK1, CDC25A, SKP2, NEDD8, CDK2, CKS1 |
| 9 | Reproduction_Progesterone-mediated oocyte maturation | 6.99E-10 | CDC25C, BUB1, MEK1(MAP2K1), Cyclin B1, Aurora-A, PLK1, c-Raf-1, GSK3 beta, Adenylate cyclase, CDC20, SOS, CDC25B, Kinase MYT1 |
| 10 | Cell cycle_ESR1 regulation of G1/S transition | 1.58E-09 | Cullin 1, CDK4, Cyclin A2, E2F4/DP1 complex, Cyclin A, Cyclin E, Skp2/TrCP/FBXW, CDC25A, SKP2, E2F4, CDK2, CKS1 |
| 11 | DNA damage_ATM/ATR regulation of G2/M checkpoint: nuclear signaling | 3.6E-09 | CDC25C, CDC18L (CDC6), Cyclin B1, Chk2, Cyclin A, DNMT1, Chk1, PLK1, GTSE1, Cyclin B, Cyclin B2, TTK, CDK2 |
| 12 | Cell cycle_Role of Nek in cell cycle regulation | 7.47E-09 | Nek2A, Tubulin beta, Tubulin gamma, Cyclin B1, TPX2, Aurora-A, PI3K cat class IA, HEC, Tubulin alpha, MAD2a, Tubulin (in microtubules) |
| 13 | DNA damage_ATM/ATR regulation of G2/M checkpoint: cytoplasmic signaling | 1.93E-08 | CDC25C, UBE2C, Cyclin B1, Chk2, PP2A regulatory, Chk1, Aurora-A, PLK1, PP1-cat, Aurora-B, CDC25A, CDC25B, 14-3-3 |
| 14 | Cell cycle_Regulation of G1/S transition (part 2) | 2.39E-08 | CDK4, Cyclin A2, E2F4/DP1 complex, DP1, p107, Cyclin A, Cyclin E, GSK3 beta, E2F4, CDK2 |
| 15 | Abnormalities in cell cycle in SCLC | 3.53E-08 | CDK4, Cyclin B1, Cyclin A, Cyclin E, Aurora-B, SKP2, E2F2, Cyclin E2, CDK2, CKS1 |
| 16 | Cell cycle_Initiation of mitosis | 1.68E-07 | CDC25C, Lamin B, Cyclin B1, PLK1, KNSL1, Cyclin B2, CDC25B, FOXM1, Kinase MYT1 |
| 17 | Cell cycle_Nucleocytoplasmic transport of CDK/Cyclins | 2.04E-07 | CDK4, Importin (karyopherin)-alpha, Cyclin B1, Cyclin A, Cyclin E, GSK3 beta, CDK2 |
| 18 | Immune response_IFN-alpha/beta signaling via PI3K and NF-κB pathways | 2.07E-07 | CDK4, I-κB, MEK1/2, I-TAC, p107, Cyclin A, p70 S6 kinases, Cyclin E, PI3K cat class IA, c-Raf-1, GSK3 beta, p107/E2F4, CDC25A, eIF4G1/3, E2F4, CDK2 |
| 19 | Translation_Regulation of EIF2 activity | 6.24E-07 | GSK3 alpha/beta, Casein kinase II, beta chain (Phosvitin), MEK1/2, Casein kinase I, PP1-cat, PI3K cat class IA, c-Raf-1, SOS, PP1-cat alpha, eIF2B5 |
| 20 | Regulation of degradation of deltaF508-CFTR in CF | 8.12E-07 | HSP90, Csp, Sti1, HSP70, Aha1, SAE1, SUMO-2, NPL4, VCP, SUMO-3 |
| 21 | Cell cycle_Influence of Ras and Rho proteins on G1/S Transition | 2.23E-06 | CDK4, MEK1(MAP2K1), Cyclin A2, DIA1, Cyclin E, PI3K cat class IA, c-Raf-1, GSK3 beta, SKP2, LIMK2, CDK2 |
| 22 | Cell cycle_Transition and termination of DNA replication | 2.31E-06 | TOP2 alpha, Ribonuclease H1, Cyclin A, MCM2, TOP2, POLD reg (p50), FEN1, CDK2 |
| 23 | Possible regulation of HSF-1/ chaperone pathway in Huntington's disease | 5.55E-06 | HSP90, GSK3 alpha/beta, PLA2, HSP70, PLK1, SUMO-2, HSP90 beta |
| 24 | Cell cycle_Regulation of G1/S transition (part 1) | 5.95E-06 | CDK4, Chk2, PP2A regulatory, Cyclin A, Cyclin E, Skp2/TrCP/FBXW, GSK3 beta, CDC25A, CDK2 |
| 25 | LRRK2 in neurons in Parkinson's disease | 1.65E-05 | AP-2 alpha subunits, HSP90, MEK1/2, GSK3 beta, MARK2, AP2A1, Tubulin (in microtubules), 14-3-3 |

**Supplementary Table 4. Pathway analysis of genes co-expressed with 26S proteasome delta subunit, non-ATPase 3 (*PSMD3*) from public breast cancer databases using the MetaCore platform (with *p*<0.01 set as the cutoff value).**

| No. | Map | *p*-Value | Network objects from active data |
|---|---|---|---|
| **1** | Cell cycle_Role of APC in cell cycle regulation | 3.21E-11 | Nek2A, CDC18L (CDC6), CDH1, Tome-1, Aurora-A, PLK1, Aurora-B, CDC25A, Cyclin B, MAD2a, ORC1L, CDK2 |
| **2** | Cell cycle_Spindle assembly and chromosome separation | 4.92E-11 | Nek2A, Importin (karyopherin)-alpha, TPX2, CSE1L, DCTN2, Aurora-A, Aurora-B, Tubulin alpha, Cyclin B, MAD2a, Separase, Tubulin (in microtubules) |
| **3** | Cell cycle_Role of Nek in cell cycle regulation | 6.49E-10 | Nek2A, Tubulin beta, Tubulin gamma, Cyclin B1, TPX2, Aurora-A, Tubulin alpha, MAD2a, Histone H1, Histone H3, Tubulin (in microtubules) |
| **4** | DNA damage_Intra S-phase checkpoint | 2.89E-09 | CDC18L (CDC6), CDH1, DTL (hCdt2), Chk2, MCM4, PP1-cat, CDC25A, MCM7, Brca1, PP1-cat alpha, MCM2, Histone H3, CDK2, GCN5, CDC45L |
| **5** | DNA damage_ATM/ATR regulation of G2/M checkpoint: cytoplasmic signaling | 1.39E-08 | UBE2C, Cyclin B1, Chk2, PP2A regulatory, Aurora-A, PLK1, PP1-cat, Aurora-B, CDC25A, Brca1, Histone H3, 14-3-3 |
| **6** | Cell cycle_Transition and termination of DNA replication | 2.28E-08 | TOP2 alpha, Brca1, TOP1, MCM2, TOP2, POLD reg (p50), FEN1, DNA ligase I, CDK2 |
| **7** | Cell cycle_Chromosome condensation in prometaphase | 5.90E-08 | CAP-H/H2, Aurora-A, Aurora-B, TOP1, Cyclin B, TOP2, Histone H1, Histone H3 |
| **8** | Cytoskeleton remodeling_Keratin filaments | 5.33E-07 | Tubulin beta, Keratin 8, Tubulin gamma 1, Keratin 18, Keratin 19, Tubulin alpha, Keratin 8/18, GRB2, Tubulin (in microtubules) |
| **9** | Transcription_Negative regulation of HIF1A function | 3.57E-06 | HSP90, Calpain 1(mu), HSP70, RUVBL2, Casein kinase I delta, Sirtuin7, HSP90 beta, MCM7, VCP, MCM2, PSMA7 |
| **10** | Cell cycle_The metaphase checkpoint | 6.04E-06 | Nek2A, Aurora-A, PLK1, Aurora-B, HZwint-1, MAD2a, Survivin, CENP-E |
| **11** | Regulation of degradation of deltaF508-CFTR in CF | 1.14E-05 | HSP90, Csp, Sti1, HSP70, Aha1, NPL4, Derlin1, VCP |
| **12** | Cell cycle_Start of DNA replication in early S phase | 2.61E-05 | CDC18L (CDC6), MCM4, MCM2, ORC1L, Histone H1, CDK2, CDC45L |
| **13** | LRRK2 in neurons in Parkinson's disease | 3.23E-05 | AP-2 alpha subunits, HSP90, MARK2, AP2A1, Tubulin (in microtubules), Beta-adaptin 2, 14-3-3 |
| **14** | DNA damage_ATM/ATR regulation of G2/M checkpoint: nuclear signaling | 3.44E-05 | CDC18L (CDC6), Cyclin B1, CDH1, Chk2, PLK1, Brca1, Cyclin B, CDK2 |
| **15** | Signal transduction_mTORC1 downstream signaling | 4.58E-05 | SCD, p70 S6 kinase2, MVK, p70 S6 kinases, UBF, SIN1, MAF1, ATG13, ULK1 |
| **16** | Apoptosis and survival_Regulation of apoptosis by mitochondrial proteins | 4.71E-05 | Calpain 1(mu), PKC-delta, Metaxin 1, Smac/Diablo, RAD9A, 14-3-3 zeta/delta, PP1-cat alpha, PP2C, LETM1, RAD9, SOD1, CDK2 |
| **17** | Regulation of lipid metabolism_Regulation of lipid metabolism via LXR, NF-Y and SREBP | 8.45E-05 | AMPK gamma subunit, SCD, FASN, LDLR, ACACA, ACLY, RARalpha |
| **18** | Translation_Regulation of EIF2 activity | 8.45E-05 | PKR, Casein kinase I, PP1-cat, H-Ras, PP1-cat alpha, eIF2AK1, GRB2 |
| **19** | DNA damage_ATM-dependent double-strand break foci | 9.86E-05 | STARING, PRMT1, NPL4, Histone H2A, Brca1, VCP, BRG1, Histone H3, GCN5 |
| **20** | Apoptosis and survival_Endoplasmic reticulum stress response pathway | 1.72E-04 | Calpain 1(mu), I-κB, TRAF2, PP1-cat, Derlin1, GRP78, PP1-cat alpha, ERP5 |
| **21** | Regulation of degradation of wtCFTR | 1.99E-04 | HSP90, Csp, NPL4, Derlin1, VCP |
| **22** | NETosis in SLE | 2.06E-04 | DNase I, Histone H2, Histone H2A, PKC, Histone H1, Histone H3 |
| **23** | SCAP/SREBP Transcriptional Control of Cholesterol and FA Biosynthesis | 2.56E-04 | ELOVL1, SCD, FASN, ERG1, MVK, ACACA, ACLY |
| **24** | Mechanisms of resistance to EGFR inhibitors in lung cancer | 2.56E-04 | HSP90, E-cadherin, H-Ras, Claudin-7, ErbB2, Survivin, GRB2 |
| **25** | Transport_Induction of Macropinocytosis | 2.96E-04 | HSP90, ARF1, BAIAP2, SHIP2, H-Ras, 14-3-3 zeta/delta, PDGF-B, PKC, RhoGDI alpha |

**Supplementary Table 5. Pathway analysis of genes co-expressed with 26S proteasome delta subunit, non-ATPase 7 (*PSMD7*) from public breast cancer databases using the MetaCore platform (with *p*<0.01 set as the cutoff value).**

| No. | Map | *p*-Value | Network objects from active data |
|---|---|---|---|
| 1 | Cell cycle_Role of APC in cell cycle regulation | 2.73E-11 | BUB1, CDH1, Geminin, Emi1, Cyclin A, Aurora-A, PLK1, PKA-cat (cAMP-dependent), Cyclin B, MAD2a, Securin, CKS1 |
| 2 | Cell cycle_ESR1 regulation of G1/S transition | 2.64E-08 | CDK4, Cyclin A2, E2F4/DP1 complex, p130, Cyclin A, ERK1/2, E2F4, ERK2 (MAPK1), CKS1, CDK6 |
| 3 | Cell cycle_Regulation of G1/S transition (part 2) | 4.24E-08 | CDK4, Cyclin A2, E2F4/DP1 complex, p130, DP1, Cyclin A, ERK1/2, E2F4, CDK6 |
| 4 | Cell cycle_Cell cycle (generic schema) | 5.29E-08 | CDK4, E2F5, p130, DP1, Cyclin A, Cyclin B, E2F4, CDK6 |
| 5 | Cell cycle_Spindle assembly and chromosome separation | 2.09E-07 | Importin (karyopherin)-alpha, Aurora-A, HEC, Tubulin alpha, Cyclin B, MAD2a, Securin, Ran, Tubulin (in microtubules) |
| 6 | Cell cycle_The metaphase checkpoint | 4.73E-07 | BUB1, SPBC25, CENP-A, Aurora-A, PLK1, HEC, HZwint-1, MAD2a, CENP-E |
| 7 | The role of aberrations in CDKN2 locus and CDK4 in familial melanoma | 6.81E-07 | CDK4, E2F4/DP1 complex, E2F5, p130, DP1, E2F5/DP1 complex, E2F4, CDK6 |
| 8 | Possible regulation of HSF-1/ chaperone pathway in Huntington's disease | 1.08E-06 | HSP90, PLA2, HSP70, HSP90 alpha, PLK1, ERK1 (MAPK3), p23 co-chaperone |
| 9 | Putative role of Estrogen receptor and Androgen receptor signaling in progression of lung cancer | 4.23E-06 | MEK1(MAP2K1), E-cadherin, p38 MAPK, ERK1 (MAPK3), G-protein alpha-i family, Caspase-3, ERK1/2, ERK2 (MAPK1), SRD5A1, 14-3-3 |
| 10 | Signal transduction_Adenosine A3 receptor signaling pathway | 6.26E-06 | HIF1A, MEK1/2, p38 MAPK, G-protein alpha-i family, G-protein alpha-i3, G-protein alpha-i2, ERK1/2, PKC, G-protein alpha-q/11 |
| 11 | Transport_RAN regulation pathway | 6.57E-06 | NTF2, NUP54, Importin (karyopherin)-alpha, RanBP1, NUP153, Ran |
| 12 | Cell cycle_Role of SCF complex in cell cycle regulation | 1.19E-05 | CDK4, p130, Emi1, Chk1, PLK1, NEDD8, CKS1 |
| 13 | NRF2 regulation of oxidative stress response | 1.71E-05 | Casein kinase II, alpha chains, MEK1(MAP2K1), Thioredoxin, PRDX1, TXNRD1, GCL reg, ERK1 (MAPK3), PKC, ERK2 (MAPK1) |
| 14 | Cell cycle_Chromosome condensation in prometaphase | 1.79E-05 | CAP-C, Cyclin A, CAP-G/G2, Aurora-A, CAP-E, Cyclin B |
| 15 | Cell cycle_Role of Nek in cell cycle regulation | 2.39E-05 | Tubulin beta, Aurora-A, HEC, Tubulin alpha, MAD2a, Ran, Tubulin (in microtubules) |
| 16 | The role of KEAP1/NRF2 pathway in skin sensitization | 2.39E-05 | HSP70, Thioredoxin, E-cadherin, TXNRD1, ERK1 (MAPK3), ERK1/2, ERK2 (MAPK1) |
| 17 | Immune response_Antigen presentation by MHC class II | 2.64E-05 | HSP90, Cathepsin L, Dectin-1, HSP90 alpha, Cathepsin V, p38 MAPK, Legumain, MARCH1, ERK1/2, HSC70, PKC, MAP1LC3B, Tubulin (in microtubules) |
| 18 | Development_S1P1 receptor signaling via beta-arrestin | 3.63E-05 | MEK1(MAP2K1), ERK1 (MAPK3), G-protein alpha-i family, G-protein alpha-i3, G-protein alpha-i2, ERK1/2, ERK2 (MAPK1) |
| 19 | Development_Regulation of telomere length and cellular immortalization | 4.43E-05 | HSP90, hnRNP C, TRF2, PTOP, hRap1, Staufen, p23 co-chaperone |
| 20 | G protein-coupled receptors signaling in lung cancer | 4.94E-05 | PGE2R4, Galpha(i)-specific peptide GPCRs, G-protein alpha-i family, TGF-alpha, PKA-cat (cAMP-dependent), Galanin, Galpha(q)-specific peptide GPCRs, CXCR4, ERK1/2, G-protein alpha-q/11 |
| 21 | HSP70 and HSP40-dependent folding in Huntington's disease | 5.30E-05 | HSP90, HSP70, HSP90 alpha, PSMD1, Hdj-2, HSC70 |
| 22 | DNA damage_ATM/ATR regulation of G2/M checkpoint: cytoplasmic signaling | 7.93E-05 | p38alpha (MAPK14), Chk1, Aurora-A, PLK1, p38 MAPK, DCK, ERK2 (MAPK1), 14-3-3 |
| 23 | HCV-dependent cytoplasmic signaling leading to HCC | 8.45E-05 | MEK1(MAP2K1), p38 MAPK, PKA-cat (cAMP-dependent), ERK1/2, PKC, ERK2 (MAPK1) |
| 24 | Non-genomic signaling of ESR2 (membrane) in lung cancer cells | 1.05E-04 | MEK1(MAP2K1), ERK1 (MAPK3), N-Ras, G-protein alpha-i family, TGF-alpha, PKA-cat (cAMP-dependent), ERK1/2, ERK2 (MAPK1) |
| 25 | Signal transduction_CXCR4 signaling via MAPKs cascades | 1.05E-04 | MEK1(MAP2K1), MEK1/2, p38 MAPK, N-Ras, G-protein alpha-i family, G-protein alpha-i2, CXCR4, ERK1/2 |

**Supplementary Table 6. Pathway analysis of genes co-expressed with 26S proteasome delta subunit, non-ATPase 10 (*PSMD10*) from public breast cancer databases using the MetaCore platform (with *p*<0.01 set as the cutoff value).**

| No. | Map | p-Value | Network objects from active data |
|---|---|---|---|
| 1 | HSP70 and HSP40-dependent folding in Huntington's disease | 9.52E-07 | HSP90, Ubiquitin, HSP70, HSP90 alpha, PSMD1, Hdj-2, HSC70 |
| 2 | DNA damage_Nucleotide excision repair | 3.58E-06 | ERCC8, ERCC6, PCNA, HMG14, Centrin-2, TFIIS, Histone H2A, E2N(UBC13), NEDD4, NEDD8 |
| 3 | CFTR folding and maturation (normal and CF) | 1.21E-05 | HSP70, Calnexin, HSP105, HSP90 alpha, Hdj-2, p23 co-chaperone |
| 4 | Immune response_Antigen presentation by MHC class II | 1.43E-05 | HSP90, Cathepsin L, 14-3-3 beta/alpha, HSP90 alpha, Cathepsin V, PI3K cat class IA, JNK(MAPK8-10), p38 MAPK, LAMP2, MARCH1, HSC70, SPPL2a |
| 5 | Regulation of degradation of deltaF508-CFTR in CF | 2.32E-05 | HSP90, Ubiquitin, HSP70, RNF4, HSP105, Hdj-2, HSC70 |
| 6 | Cell cycle_Role of SCF complex in cell cycle regulation | 3.89E-05 | Ubiquitin, p130, Emi1, Skp2/TrCP/FBXW, Wee1, NEDD8 |
| 7 | Immune response_BAFF-induced non-canonical NF-kB signaling | 4.77E-05 | Ubiquitin, SUMO-1, UBE1C, Skp2/TrCP/FBXW, E2N(UBC13), NEDD8 |
| 8 | Development_Positive regulation of WNT/Beta-catenin signaling in the cytoplasm | 5.15E-05 | PP2C alpha, GSKIP, SIAH1, HSP105, JNK(MAPK8-10), SMAD4, PP2A catalytic, RNF146, 14-3-3 |
| 9 | Tricarbonic acid cycle | 6.45E-05 | SDHA, SUCLG1, SDHB, CISY, SUCB1, IDH3B, DLDH, SCS-A |
| 10 | Role of XBP1 protein in multiple myeloma | 6.70E-05 | SERP1, DnaJB9, PSMA6, GRP78, ERP5 |
| 11 | Role of GIP in pathogenesis of type 2 diabetes | 7.06E-05 | Ubiquitin, RAP-1A, p38alpha (MAPK14), MEK1/2, JNK(MAPK8-10), p38 MAPK, PP2A catalytic |
| 12 | Possible regulation of HSF-1/ chaperone pathway in Huntington's disease | 8.63E-05 | HSP90, HSP70, HSP90 alpha, JNK(MAPK8-10), p23 co-chaperone |
| 13 | DNA damage_ATM/ATR regulation of G2/M checkpoint: cytoplasmic signaling | 1.39E-04 | JAB1, p38alpha (MAPK14), p38 MAPK, JNK2(MAPK9), DCK, PP2A catalytic, 14-3-3 |
| 14 | Transcription_Negative regulation of HIF1A function | 1.57E-04 | HSP90, PRDX4, Ubiquitin, HSP70, FBXW7, LAMP2, Elongin C, HSC70 |
| 15 | Proteolysis_Role of Parkin in the Ubiquitin-Proteasomal Pathway | 1.70E-04 | SIAH1, HSP70, FBXW7, UBC7, Tubulin alpha |
| 16 | G-protein signaling_G-Protein alpha-12 signaling pathway | 1.90E-04 | MEK1(MAP2K1), RAP-1A, 14-3-3 beta/alpha, PI3K cat class IA, JNK(MAPK8-10), p38 MAPK |
| 17 | Immune response_HSP60 and HSP70/ TLR signaling pathway | 2.00E-04 | Ubiquitin, HSP70, I-kB, MEK1/2, JNK(MAPK8-10), p38 MAPK, E2N(UBC13) |
| 18 | Translation_Regulation of EIF4F activity | 2.00E-04 | MEK1(MAP2K1), eIF4H, PI3K cat class IA, p38 MAPK, PP2A catalytic, RHEB2, eIF4E |
| 19 | Development_Glucocorticoid receptor signaling | 2.09E-04 | HSP90, SUMO-1, HSP70, NCOA2 (GRIP1/TIF2), p23 co-chaperone |
| 20 | G-protein signaling_Ras family GTPases in kinase cascades | 2.54E-04 | MEK1(MAP2K1), RAP-1A, p38alpha (MAPK14), JNK(MAPK8-10), p38 MAPK |
| 21 | Immune response_TLR5, TLR7, TLR8 and TLR9 signaling pathways | 3.15E-04 | Ubiquitin, I-kB, MEK1/2, PI3K cat class IA, JNK(MAPK8-10), p38 MAPK, E2N(UBC13) |
| 22 | Immune response_IL-33 signaling pathway | 3.15E-04 | Ubiquitin, p38alpha (MAPK14), I-kB, MEK1/2, PI3K cat class IA, Histone H2A, JNK(MAPK8-10) |
| 23 | Signal transduction_AKT signaling | 3.83E-04 | HSP90, PCNA, I-kB, PI3K cat class IA, PP2A catalytic, RHEB2 |
| 24 | ESR1 (membrane) 36 kDa isoform signaling in breast cancer | 3.83E-04 | HSP90, E-cadherin, MEK1/2, PI3K cat class IA, JNK(MAPK8-10), CXCR4 |
| 25 | DNA damage_p53 activation by DNA damage | 3.89E-04 | TTC5 (Strap), p38alpha (MAPK14), 14-3-3 theta, JNK(MAPK8-10), p38 MAPK, PP2A catalytic, 14-3-3 |

**Supplementary Table 7. Pathway analysis of genes co-expressed with 26S proteasome delta subunit, non-ATPase 12 (*PSMD12*) from public breast cancer databases using the MetaCore platform (with *p*<0.01 set as the cutoff value).**

| No. | Map | *p*-Value | Network objects from active data |
|---|---|---|---|
| 1 | Cell cycle_Role of APC in cell cycle regulation | 2.62E-23 | Nek2A, BUB1, CDC18L (CDC6), CDH1, Tome-1, Geminin, Emi1, Cyclin A, Aurora-A, PLK1, Aurora-B, CDC25A, CDC20, SKP2, Cyclin B, MAD2a, Securin, ORC1L, CDK2, CKS1 |
| 2 | Cell cycle_The metaphase checkpoint | 3.68E-17 | Nek2A, INCENP, BUB1, SPBC25, CENP-A, Aurora-A, PLK1, Aurora-B, HEC, CDCA1, CDC20, HZwint-1, CENP-F, MAD2a, Survivin, CENP-E, AF15q14 |
| 3 | DNA damage_Intra S-phase checkpoint | 2.96E-15 | PCNA, CDC18L (CDC6), BLM, CDH1, FANCD2, DTL (hCdt2), Histone H2AX, Chk2, MCM4, MCM3, Cyclin A, Chk1, FANCI (KIAA1794), CDC25A, MCM7, MCM10, CDC7, MCM2, Histone H3, CDK2, CDC45L |
| 4 | Cell cycle_Spindle assembly and chromosome separation | 5.66E-15 | Nek2A, Importin (karyopherin)-alpha, TPX2, CSE1L, Aurora-A, KNSL1, Aurora-B, HEC, CDC20, Tubulin alpha, Cyclin B, MAD2a, Separase, Securin, Tubulin (in microtubules) |
| 5 | DNA damage_ATM/ATR regulation of G2/M checkpoint: nuclear signaling | 1.36E-12 | CDC25C, WDHD1, CDC18L (CDC6), Cyclin B1, CDH1, HSF1, Histone H2AX, Chk2, Cyclin A, Chk1, PLK1, Cyclin B, Cyclin B2, TTK, CDK2 |
| 6 | Cell cycle_Start of DNA replication in early S phase | 2.27E-12 | CDC18L (CDC6), Geminin, MCM4, MCM3, Cyclin E, MCM10, ORC6L, MCM4/6/7 complex, CDC7, MCM2, ORC1L, CDK2, CDC45L |
| 7 | Cell cycle_Cell cycle (generic schema) | 3.57E-12 | CDC25C, CDK4, E2F5, p107, Cyclin A, Cyclin E, CDC25A, Cyclin B, E2F2, CDC25B, CDK2 |
| 8 | Cell cycle_Chromosome condensation in prometaphase | 3.57E-12 | INCENP, CAP-C, Cyclin A, CNAP1, CAP-G/G2, Aurora-A, CAP-D2/D3, Aurora-B, Cyclin B, TOP2, Histone H3 |
| 9 | DNA damage_ATM/ATR regulation of G2/M checkpoint: cytoplasmic signaling | 1.07E-11 | CDC25C, UBE2C, Cyclin B1, JAB1, BORA, Chk2, Chk1, Aurora-A, PLK1, Aurora-B, CDC25A, DCK, CDC25B, Histone H3, 14-3-3 |
| 10 | Abnormalities in cell cycle in SCLC | 1.25E-11 | CDK4, PCNA, Cyclin B1, Cyclin A, Cyclin E, Aurora-B, SKP2, E2F2, Histone H3, Cyclin E2, CDK2, CKS1 |
| 11 | Cell cycle_Role of SCF complex in cell cycle regulation | 2.83E-10 | CDK4, Emi1, Cyclin E, Skp2/TrCP/FBXW, Chk1, PLK1, CDC25A, SKP2, NEDD8, CDK2, CKS1 |
| 12 | Cell cycle_Role of Nek in cell cycle regulation | 9.73E-10 | Nek2A, Tubulin beta, Tubulin gamma, Cyclin B1, TPX2, Aurora-A, HEC, Tubulin alpha, MAD2a, Histone H3, Tubulin (in microtubules) |
| 13 | Cell cycle_Initiation of mitosis | 1.59E-09 | CDC25C, Lamin B, Cyclin B1, PLK1, KNSL1, Cyclin B2, CDC25B, FOXM1, Kinase MYT1, Histone H3 |
| 14 | Cell cycle_ESR1 regulation of G1/S transition | 2.91E-09 | CDK4, Cyclin A2, NCOA3 (pCIP/SRC3), Cyclin A, Cyclin E, Skp2/TrCP/FBXW, CDC25A, SKP2, CRM1, CDK2, CKS1 |
| 15 | Cell cycle_Nucleocytoplasmic transport of CDK/Cyclins | 5.40E-08 | CDK4, Importin (karyopherin)-alpha, Cyclin B1, Cyclin A, Cyclin E, CRM1, CDK2 |
| 16 | DNA damage_ATM/ATR regulation of G1/S checkpoint | 4.62E-07 | CDK4, PCNA, Histone H2AX, Chk2, Cyclin A, Cyclin E, Chk1, CDC25A, CDK2, RFWD3 |
| 17 | Mitogenic action of Estradiol / ESR1 (nuclear) in breast cancer | 1.01E-06 | CDK4, NCOA3 (pCIP/SRC3), WIP1, Cyclin E, SGOL2, CDC25A, Cyclin E2, CDK2 |
| 18 | Possible regulation of HSF-1/ chaperone pathway in Huntington's disease | 1.53E-06 | HSP90, PLA2, HSP70, HSF1, HSP90 alpha, PLK1, p23 co-chaperone |
| 19 | Cell cycle_Role of 14-3-3 proteins in cell cycle regulation | 2.18E-06 | CDC25C, Chk2, 14-3-3 theta, Chk1, CDC25A, 14-3-3 zeta/delta, CDC25B |
| 20 | Cell cycle_Sister chromatid cohesion | 3.06E-06 | PCNA, Rad21, Cyclin B, DCC1, Separase, Securin, Histone H3 |
| 21 | DNA damage_Nucleotide excision repair | 5.95E-06 | ERCC6, PCNA, DTL (hCdt2), EZH2, UFD1, Histone H2A, DNA polymerase kappa, Histone H2B, NEDD8, Histone H4, Histone H3 |
| 22 | Cell cycle_Transition and termination of DNA replication | 7.60E-06 | TOP2 alpha, PCNA, Cyclin A, MCM2, TOP2, FEN1, CDK2 |
| 23 | Regulation of degradation of deltaF508-CFTR in CF | 1.50E-05 | HSP90, Csp, Sti1, HSP70, SAE1, HSP105, UFD1, Derlin1 |
| 24 | Reproduction_Progesterone-mediated oocyte maturation | 1.83E-05 | CDC25C, BUB1, Cyclin B1, Aurora-A, PLK1, CDC20, CDC25B, Kinase MYT1 |
| 25 | Immune response_IFN-alpha/beta signaling via PI3K and NF-kB pathways | 2.03E-05 | CDK4, PCNA, 4E-BP1, p107, Cyclin A, Cyclin E, p19, DHFR, CDC25A, eIF4E, CDK2, ISG15 |

**Supplementary Table 8. Pathway analysis of genes co-expressed with 26S proteasome delta subunit, non-ATPase 14 (PSMD14) from public breast cancer databases using the MetaCore platform (with p<0.01 set as the cutoff value).**

| No. | Map | *p*-Value | Network objects from active data |
|---|---|---|---|
| 1 | Cell cycle_The metaphase checkpoint | 4.46E-14 | Nek2A, BUB1, SPBC25, CENP-A, Aurora-A, PLK1, Aurora-B, HEC, CDCA1, HZwint-1, MAD2a, Survivin, CENP-H, CENP-E, AF15q14 |
| 2 | Cell cycle_Role of APC in cell cycle regulation | 3.39E-12 | Nek2A, BUB1, Tome-1, Geminin, Emi1, Cyclin A, Aurora-A, PLK1, Aurora-B, Cyclin B, MAD2a, Securin, CKS1 |
| 3 | Cell cycle_Spindle assembly and chromosome separation | 5.44E-12 | Nek2A, Importin (karyopherin)-alpha, TPX2, CSE1L, Aurora-A, KNSL1, Aurora-B, HEC, Tubulin alpha, Cyclin B, MAD2a, Securin, Tubulin (in microtubules) |
| 4 | DNA damage_ATM/ATR regulation of G2/M checkpoint: cytoplasmic signaling | 1.68E-11 | UBE2C, Cyclin B1, JAB1, 14-3-3 gamma, BORA, Chk2, Chk1, Aurora-A, PLK1, PP1-cat, Aurora-B, DCK, Nucleolysin TIAR, Histone H3, 14-3-3 |
| 5 | Cell cycle_Role of Nek in cell cycle regulation | 2.23E-08 | Nek2A, Tubulin beta, Cyclin B1, TPX2, Aurora-A, HEC, Tubulin alpha, MAD2a, Histone H3, Tubulin (in microtubules) |
| 6 | DNA damage_Intra S-phase checkpoint | 5.98E-08 | PCNA, DTL (hCdt2), Chk2, PP1-cat gamma, Cyclin A, RIF1, Claspin, Chk1, FANCI (KIAA1794), PP1-cat, MCM10, CDC7, Histone H3, CDC45L |
| 7 | Cell cycle_Chromosome condensation in prometaphase | 1.01E-07 | Cyclin A, CAP-G/G2, Aurora-A, Aurora-B, CAP-E, Cyclin B, TOP2, Histone H3 |
| 8 | DNA damage_G2 checkpoint in response to DNA mismatches | 1.71E-07 | PCNA, MutSalpha complex, Chk2, MSH6, PMS1, Claspin, Chk1, EXO1, MSH2 |
| 9 | DNA damage_ATM/ATR regulation of G2/M checkpoint: nuclear signaling | 7.73E-07 | Cyclin B1, Chk2, Ku70, Cyclin A, Claspin, Chk1, PLK1, Cyclin B, Cyclin B2, TTK |
| 10 | Regulation of degradation of deltaF508-CFTR in CF | 1.98E-06 | Csp, HSP70, Aha1, HSP105, SUMO-2, Derlin1, UCHL1, Hdj-2, HSC70 |
| 11 | Cell cycle_Sister chromatid cohesion | 3.77E-06 | PCNA, Rad21, Cyclin B, DCC1, RFC3, Securin, Histone H3 |
| 12 | Oxidative stress_Role of ASK1 under oxidative stress | 4.54E-06 | HPK38, SOD2, UNRIP, 14-3-3 gamma, Thioredoxin, PRDX1, MT-TRX, Glutaredoxin, SOD1, 14-3-3 |
| 13 | Cell cycle_Initiation of mitosis | 9.36E-06 | Cyclin B1, Nucleolin, PLK1, KNSL1, Cyclin B2, FOXM1, Histone H3 |
| 14 | Transport_RAN regulation pathway | 1.06E-05 | NUP54, SUMO-1, Importin (karyopherin)-alpha, NUP58, RanBP1, CRM1 |
| 15 | Abnormalities in cell cycle in SCLC | 2.05E-05 | PCNA, Cyclin B1, Cyclin A, Aurora-B, Histone H3, Cyclin E2, CKS1 |
| 16 | Possible regulation of HSF-1/ chaperone pathway in Huntington's disease | 2.87E-05 | PLA2, HSP70, PLK1, SUMO-2, Calmodulin, p23 co-chaperone |
| 17 | Microsatellite instability in gastric cancer | 3.85E-05 | PCNA, MutSalpha complex, MSH6, PMS1, EXO1, MSH2 |
| 18 | CFTR folding and maturation (normal and CF) | 6.59E-05 | Csp, HSP70, Aha1, HSP105, Hdj-2, p23 co-chaperone |
| 19 | Release of pro-inflammatory mediators and elastolytic enzymes by alveolar macrophages in COPD | 1.66E-04 | MMP-12, Cathepsin L, MMP-1, IL-8, IP10, HDAC2 |
| 20 | Reproduction_Progesterone-mediated oocyte maturation | 1.83E-04 | BUB1, MEK1(MAP2K1), Cyclin B1, Aurora-A, PLK1, PKA-reg (cAMP-dependent), G-protein alpha-i family |
| 21 | Cell cycle_Role of SCF complex in cell cycle regulation | 2.04E-04 | Emi1, Chk1, PLK1, RING-box protein 1, NEDD8, CKS1 |
| 22 | Apoptosis and survival_Granzyme A signaling | 2.49E-04 | Ku70/80, NDPK A, Ku80, HMGB2, Ku70, Histone H3 |
| 23 | DNA damage_Mismatch repair | 3.61E-04 | PCNA, MutSalpha complex, MSH6, EXO1, MSH2, Histone H3 |
| 24 | Signal transduction_MIF signaling pathway | 5.04E-04 | MEK1/2, PRDX1, SFK, IL-8, GCL reg, G-protein alpha-i family, CXCR4, SPPL2a |
| 25 | Microsatellite instability in colorectal cancer | 5.09E-04 | PCNA, MutSalpha complex, Beta-2-microglobulin, MSH6, EXO1, MSH2 |
